# Molecular Mechanism of ATP Hydrolysis Catalyzed by p97: A QM/MM Study

**DOI:** 10.1021/acs.jctc.5c00928

**Published:** 2025-09-19

**Authors:** Judit Katalin Szántó, Andreas Hulm, Christian Ochsenfeld

**Affiliations:** † Chair of Theoretical Chemistry, Department of Chemistry, University of Munich (LMU), Butenandtstr. 5, D-81377 München, Germany; ‡ Max Planck Institute for Solid State Research, Heisenbergstr. 1, D-70569 Stuttgart, Germany

## Abstract

A computational study of p97/VCP ATPase using hybrid quantum mechanics/molecular mechanics (QM/MM) simulations is presented that explores the conformational landscape of the active site and hydrolysis-competent states of the crystallographic water molecules. Our investigation focuses on the reaction mechanism, particularly the events of the rate-determining first reaction step, which we study using extensive sampling with the path well-tempered metadynamics extended-system adaptive biasing force (WTM-eABF) enhanced sampling method. We identify the highly conserved glutamate (Glu305) from the Walker B motif as a catalytic base that activates the lytic water molecule for nucleophilic attack on the γ-phosphate in the first reaction step, while the final product is formed in a second step that involves proton transfer and rearrangements in the Mg^2+^ coordination sphere. We show that phosphate bond formation and breakage occur concertedly in the first reaction step. The findings gained through versatile QM/MM approaches are validated against recent cryo-EM and NMR data for the post-hydrolysis protein state, elucidating the role of amino acids from conserved motifs across the AAA+ protein family. To the best of our knowledge, this is the first *in silico* exploration of ATP hydrolysis in p97/VCP or any other AAA+ protein.

## Graphical abstract

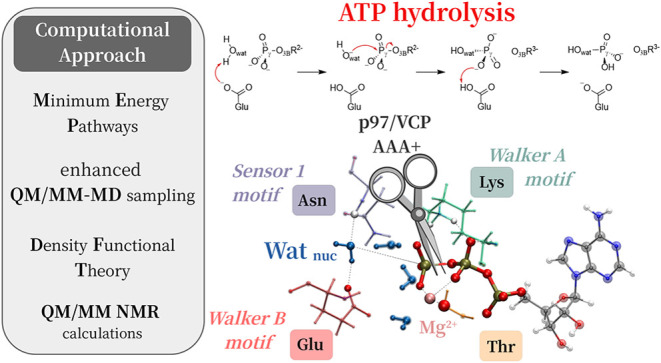

## 1 Introduction

The conversion of chemical energy in the form of ATP to exert mechanical force is one of the fundamental riddles of biochemistry. An example is p97, also known as valosin-containing protein (VCP), a hexameric motor complex and member of the AAA+ (ATPases associated with diverse cellular activities) protein superfamily that binds, hydrolyzes, and releases ATP to regulate various cellular pathways.^1^ Extensive research has been carried out on the conformational changes of the global p97 protein structure during the ATPase cycle,^2–8^ but no previous study has elucidated the molecular mechanism of ATP hydrolysis catalyzed by p97. ATP hydrolysis in solution can proceed through multiple pathways,^9,10^ and the conformational landscape becomes even more complex at the active site of a protein. Despite their functional diversity, nucleoside triphosphate (NTP) hydrolyzing enzymes (NTPase proteins) often share a common nucleotide binding fold. For instance, P-loop NTPases use a highly conserved loop to bind and efficiently hydrolyze nucleotides.^11^ Here, computer simulations that capture protein structure and dynamics using a hybrid QM/MM framework can provide full atomic details at high temporal resolution and have, in several studies, elucidated the catalytic mechanism of P-loop NTPases to which p97 belongs. For example, recent QM/MM studies on Ras-GTPases—whose malfunction drives many cancers—have revealed how oncogenic mutations alter the catalytic activity^12^ and uncovered how the structural complexity of the active site, involving different side-chain tautomers, facilitates phosphate hydrolysis.^13^

Given the complexity of enzyme-catalyzed reactions, a comprehensive understanding of factors such as the roles of amino acids, water molecules, and metal ions at the active site is essential and guides the computational exploration of the reaction mechanism. Therefore, we build on insights gained from the existing literature on ATPases and GTPases,^14^ as well as kinetic and mutational studies on the AAA+ protein family and other P-loop NTPases to which p97 belongs. For our theoretical study, amino acids that are close to the substrate and whose mutations affect nucleotide binding or ATP hydrolysis rates are crucially important. These include glutamate (Glu305) from the Walker B motif, a highly conserved residue in the nucleotide binding pocket of several AAA+ proteins.^1,15–17^ Upon mutation to glutamine, ATP binding is preserved, but hydrolysis is hindered^15,18–20^ in various members of this protein family. More precisely, experimental findings in p97 show that this mutation leads to a 20-fold decrease in enzymatic activity,^20^ suggesting that it must play a crucial role in the reaction mechanism of ATP hydrolysis.

Another important feature of the AAA+ family is the Sensor 1 motif (typically asparagine, serine, threonine, or aspartate), which was hypothesized to help orient the water molecule for the nucleophilic attack.^15,19,21–24^ Experimental evidence from kinetic studies on AAA+ Sensor 1 mutants revealed decreased catalytic activity after mutating the Sensor 1 unit,^25,26^ the typical mutation being asparagine to alanine.^1^ Other amino acids of high relevance are the conserved arginine residues,^24,27^ which, as positively charged residues, are thought to stabilize the transition state^28^ and help in the intersubunit communication.^3^ R359 and R362 residues are also called *trans*-acting arginine fingers, as they are located at the subunit interface, extending from one subunit into the active site of the neighboring one. Furthermore, a recent high-resolution cryo-EM study^8^ showed that threonine (Thr252) from the P-loop of p97 plays a key role in coordinating the Mg^2+^ ion, which neutralizes negative charges of the nucleotide’s phosphate groups. The Mg^2+^ ion is also an important protagonist in ATP catalysis, as it has previously been shown that the presence of metal ions at the active site can alter the reaction mechanism of phosphate ester hydrolysis.^10,29,30^ The full hexameric p97 complex and key residues of the active site are shown in Figure 1.

**Figure 1:**
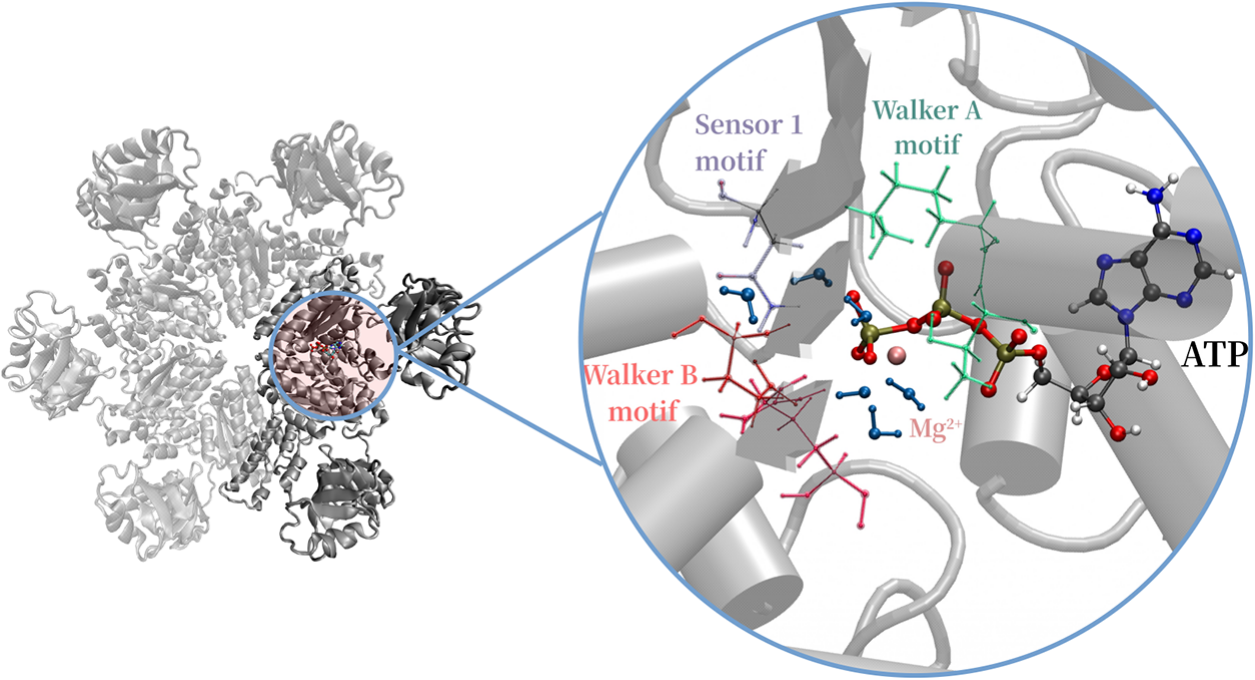
Left: Structure of the hexameric p97 featuring the N-terminal domain and the D1 nucleotide binding domain (PDB 4KO8 ^31^). Two adjacent subunits are highlighted in dark gray, which are selected for our computations. Right: schematic representation of the binding site with ATP, the Mg^2+^ ion, and crystallographic water molecules. Walker A, B, and the Sensor 1 motif, as highly conserved regions across AAA+ proteins, are highlighted with colors.

In addition to amino acids and metal ions, buried water molecules contribute to the mechanistic complexity of ATP hydrolysis. Previous QM/MM studies of other P-loop NTPases have shown that multiple water molecules can participate in the reaction, posing challenges for the computational exploration of reaction pathways. In ABC transporters, a single water molecule was found to be sufficient for ATP hydrolysis.^32^ In contrast, myosin, kinesin, and F1-ATPase require multiple water molecules. In myosin, two catalytic water molecules were found: an attacking and a helping water, which are positioned by a dense hydrogen bonding network.^33^ ATP hydrolysis in F1-ATPase occurs with the help of three water molecules, which are directly involved in the reaction process.^34^ This raises the important question of how many water molecules are involved in the catalytic mechanism of p97.

Our interest lies not only in gaining mechanistic insight but also in understanding how ^31^P chemical shifts evolve during hydrolysis. This is motivated by our previous study,^35^ where we observe a drastic change in the chemical shifts of the P_β_ nucleus for pre- and post-hydrolysis protein states, which can only fully be explained by direct investigation of the mechanism of p97.

In this work, we present a detailed investigation of the p97 reaction mechanism, using extended QM/MM calculations. For this purpose, we followed a three-step computational workflow. We start by exploring possible reaction mechanisms in the active site by testing reactions of nearby water molecules. Second, the reaction paths that lead to stable intermediates are optimized to obtain minimum energy pathways. Lastly, after a thorough benchmark of the reliability of the QM/MM setup, enhanced sampling MD simulations are performed to obtain accurate reaction and activation free energies of the rate-limiting step. We discuss the structural rearrangements at the active site during product formation and finally predict NMR chemical shifts along the reaction pathway, always thoroughly relating our findings to the experimental data. In this way, we aim to provide a complete picture of ATP hydrolysis in p97 and similar members of the AAA+ protein family.

## 2 Methods

For the QM/MM study on the reaction mechanism, we build on insights gained from our recent computational study^35^ on p97 ATPase, as well as experimental NMR,^20^ cryo-EM, and MM-MD studies^8^ on this protein. The crystal structure (PDB 4KO8 ^31^) of p97 originally contains the hydrolysis-resistant ATPγS at the active site, which was transformed into ATP, followed by QM/MM structure optimizations, and served as educt structure and starting point for exploring the reactivity. The conversion of ATPγS to ATP, system preparation, protonation state assignment of titratable groups, and equilibration of the ATP-bound p97 protein were performed by Shein et al.,^8^ who provided us with the resulting equilibrated educt structure. Compared to the hydrolysis-resistant substrate analogue (ATPγS), the presence of the true substrate (ATP) at the active site prompts the reorientation of key protein residues such as the *trans*-acting R359 and R362 arginine fingers and Glu305 (see Figure S2 in the Supporting Information (SI)). The functional p97 machine assembles from six identical subunits, also called protomers, from which we chose two neighboring protein subunits as subsystem with ca. 30000 atoms for our simulations (see Figure 1). Events such as substrate binding, inorganic phosphate release, and ADP unbinding involve complex conformational changes in the global structure of p97,^20^ and for the *in silico* study of these steps, intersubunit communication must be explicitly modeled, requiring analysis of the entire hexamer. However, our focus is on the chemical mechanism of ATP hydrolysis; therefore, only a subsystem comprising two neighboring subunits was selected for our study.

All QM/MM calculations were performed using the PBEh-3c^36^ DFT functional on 120–160 QM atoms, the rest of the system being described by the MM part using the Amber ff14Sb.^37^ Before choosing the DFT functional used for the QM/MM calculations, we performed a DFT functional benchmark study on the minimum energy pathways. Here, we observed a good agreement within 1–2 kcal/mol between the efficient PBEh-3c/def2-mSVP method and the DLPNO–CCSD­(T)/def2-QZVP^38,39^ coupled-cluster approximation. Therefore, we decided to use the PBEh-3c hybrid DFT functional, which was developed for efficient geometry optimizations and reaction energy evaluations in large molecular systems using a smaller DZ basis set to balance accuracy and cost. The influence of different DFT functionals and basis sets is presented in Section 4 of the SI.

For the QM/MM calculations, the FermiONs++ ^40–42^ quantum chemistry program package in combination with the LibXC^43^ library of exchange-correlation functionals, the OpenMM^44,45^ library, and the Adaptive-Sampling Python package^46,47^ were employed.

FermiONs++ enables hybrid DFT applications on extended biomolecular systems, due to its efficient implementations of the sn-LinK^48,49^ and RI-J^50^ methods, and also supports the linear-scaling computation of NMR chemical shieldings.^51^ QM/MM interactions were treated with an additive scheme using electrostatic embedding,^52^ and QM/MM NMR calculations were conducted at the B97–2/pcSseg-2 ^53,54^ level of theory. Complete computational details on the used methods and benchmark studies are provided in the Supporting Information, while in the following, a brief summary of the employed workflow is given.

Our methodology is based on the steps, as summarized in the overview above (Figure 2), followed by the final relation of the results to experimental studies. We begin the initial exploration using adiabatic mapping (AM) to test the nucleophilic attack of crystallographic water molecules close to the P_γ_ and P_β_ atoms of the ATP molecule. AM pathways were obtained from a sequence of energy minimizations along selected reaction coordinates. It is important to note that reaction coordinates involve only the forming of the O_Wat_-P_γ_ and/or dissociating P_γ_-O_3B_ distances, while water protons migrate to the best suited acceptor in an unbiased fashion. In our workflow, AM was used solely to identify the next local minimum on the potential energy surface (PES). Once a minimum was found, the Nudged Elastic Band (NEB) method^55^ was applied to determine the minimum energy path (MEP) between the optimized metastable states. Next, we carried out a thorough benchmark study to determine the appropriate QM region size, as shown in Sections S5–S6 of the SI.

**Figure 2:**
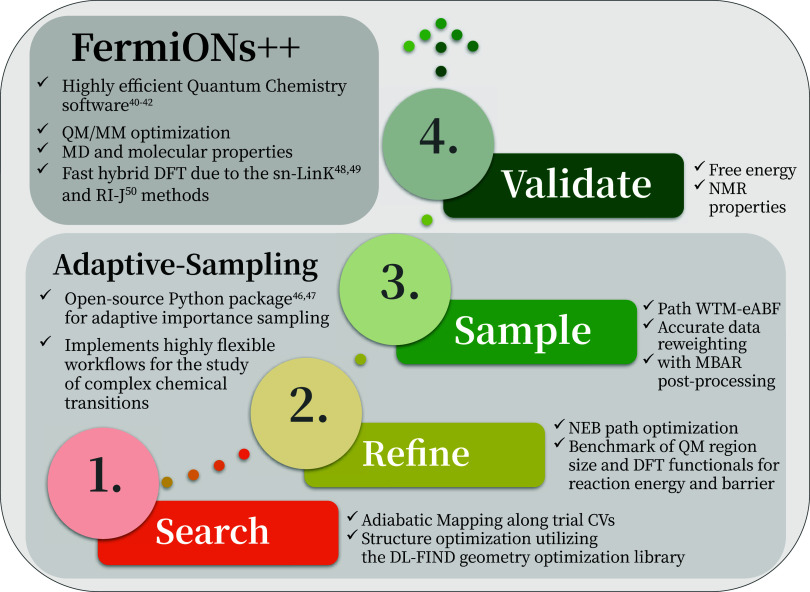
Workflow overview, which builds on the FermiONs++ ^40–42^ quantum chemistry software and the Adaptive-Sampling Python package.^46,47^

To obtain accurate activation and reaction free energies, we explore the potential of mean force (PMF) (i.e., free energy surface) of the rate-determining step by sampling the underlying PES. This is the most computationally demanding step of our approach, as the time step is 0.5 fs, and the total sampling time exceeds 2 ns. This extensive sampling was achieved through the high efficiency of our in-house FermiONs++ ^40–42^ quantum chemistry code, enabling the computation of statistically robust free energy profiles of the rate-limiting reaction steps.

The choice of the collective variable (CV), which provides a measure of the reaction progress, is crucial for successful importance sampling simulations, as a poor selection of the CV can drastically influence the obtained activation free energy.^56^ Therefore, we used the NEB optimized reaction pathway as a path collective variable (PCV)^57^ for sampling with the well-tempered metadynamics extended-system adaptive biasing force method (WTM-eABF).^58–60^ We choose WTM-eABF over static sampling methods like Umbrella Sampling (US), because it facilitates the free diffusion of the system along CVs, resulting in the broad and reliable sampling of transition pathways.^61^ The CV space for the PCV was defined *a priori* by selecting breaking and forming bond distances involved in the corresponding step, ensuring that the CVs clearly differentiate key states and remain minimal in number. For further details on the parameters used in the QM/MM-MD sampling, see Section S7 of the SI. Finally, we validate our results by comparison with recent cryo-EM^8^ and experimental NMR data,^20^ which capture the ADP.P_i_ post-hydrolysis protein state prior to the release of inorganic phosphate (P_i_).

## 3 Results and Discussion

The X-ray structure with PDB 4KO8 ^31^ reveals buried water molecules at the active site (see Figure S3 of the SI), which form a highly conserved and integral part of the structure of the p97 protein. In the computational modeling of the reaction mechanism, the first challenge is identifying the catalytic water molecule responsible for cleaving the phosphate group as well as the role of key catalytic amino acids such as conserved Glu305 and Sensor 1 Asn348 in the process, as discussed in Sections 3.1–3.3, respectively. After the initial nucleophilic attack, the final product is built in a second reaction step that involves proton transfer and rearrangement of the Mg^2+^ coordination sphere (Section 3.4). Finally, both reaction pathways are compared to solid-state NMR measurements (Section 3.5).

### 3.1 Walker B Glu305 Acts as a Catalytic Base

We started by testing the influence of differently oriented crystallographic water molecules on the activation barrier of the first step in ATP hydrolysis. Close to the reaction center, we observe the same number of water molecules as in the crystal structure, occupying well-defined locations throughout the long-timescale MD simulation of the educt structure reported by Shein et al.,^8^ as shown in Figures S10 and S11 of the SI. We identified three water molecules as likely candidates for the nucleophilic attack, based on their proximity to the P_γ_ atom or to a suitable proton acceptor. In the first step of phosphate hydrolysis, the H^+^ of the lytic water molecule is transferred to a nearby proton acceptor, OH^–^ attacks the P_γ_, and the P_γ_-O_3B_ bond breaks. Our exploration using adiabatic mappings (see Section S2 of the SI) shows that the proton can be accepted by either a protein group acting as a base (base-assisted) or by the phosphate itself (substrate-assisted). Additionally, one or two water molecules can be involved. An overview of the obtained reaction mechanisms is given in Figure 3.

**Figure 3:**
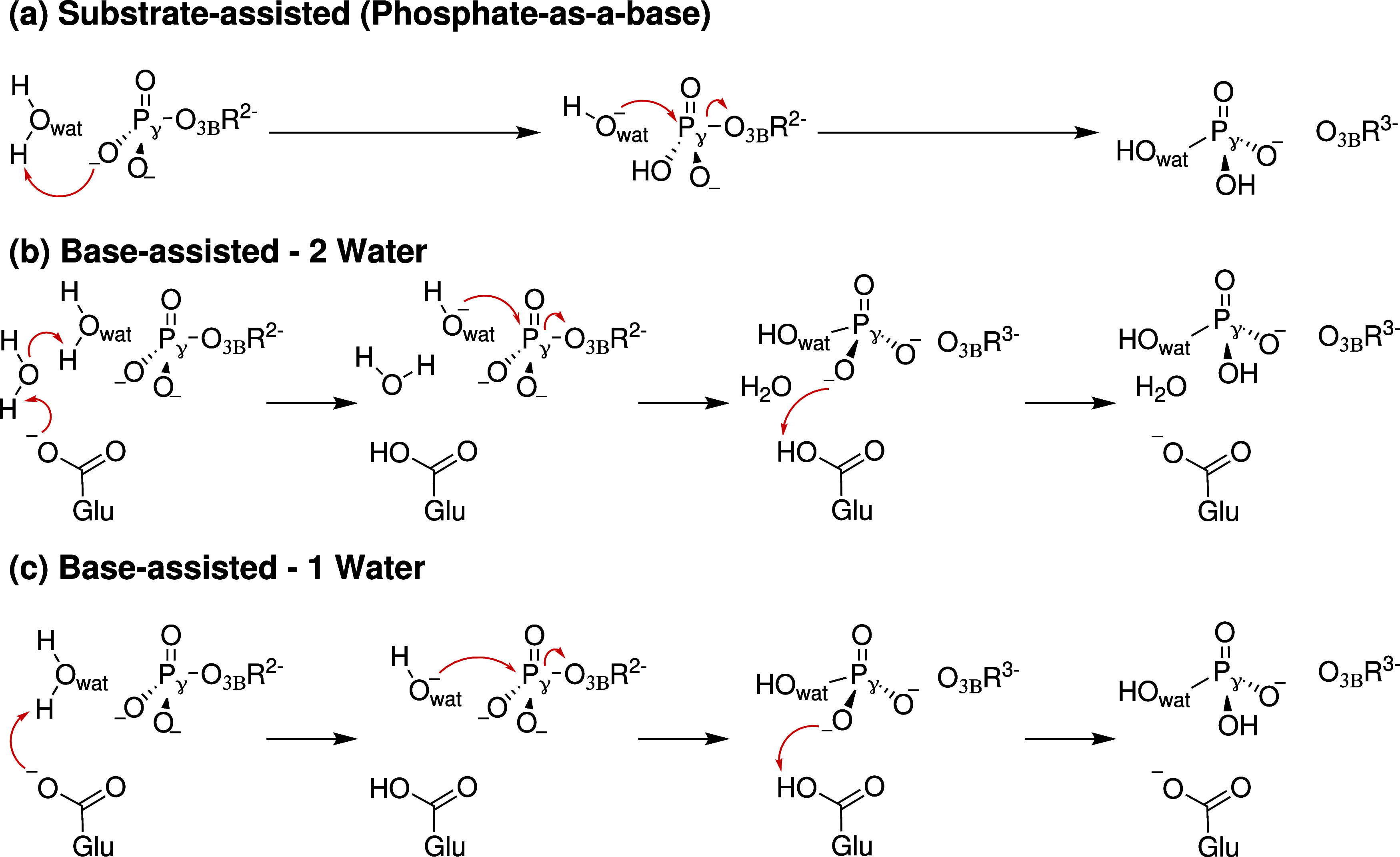
Observed reaction mechanisms using adiabatic mapping.

From the MEPs presented in Figure 4, which are obtained by NEB optimization of AM reaction pathways, we conclude that the base-assisted (Glu305) mechanisms with the participation of a single water molecule are more favorable than the two water or the substrate-assisted (phosphate-as-a-base) mechanisms, whose activation energy barrier is more than 20 kcal/mol higher. For the Glu305-assisted, single water path, we identify two distinct reaction channels (channel A and B), as further discussed below. Furthermore, we can conclude that a single water molecule can hydrolyze ATP in p97. However, for enzymes where the catalytic base is positioned farther away from the P_γ_ or the nucleophilic water molecule, the proton transfer may require a longer pathway, potentially involving one or more bridging water molecules.^33,62,63^ That the substrate-assisted mechanism is not feasible is supported by previous QM/MM studies^32,64^ on P-loop NTPases, which likewise identify the base-assisted pathway as the more favorable route—reported to be 10 kcal/mol^32^ or 26 kcal/mol lower in energy.^64^

**Figure 4:**
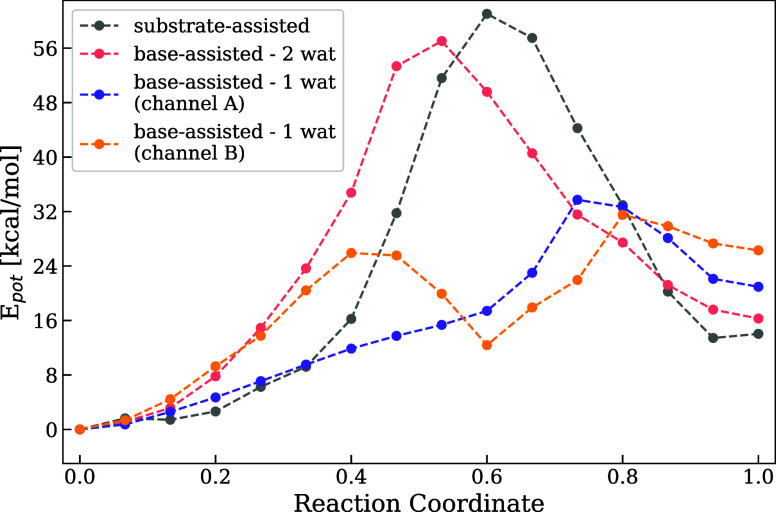
First step of ATP hydrolysis. Minimum energy profiles (MEPs) were obtained for the substrate- and base-assisted reaction mechanisms.

The observation that glutamate plays a direct role in ATP hydrolysis aligns well with experimental findings in p97, which show that mutating this conserved glutamate to glutamine alters the catalytic rate constant and abolishes ATP hydrolysis,^31,65^ more specifically resulting in a 20-fold decrease in enzymatic activity.^20^ Additional support for the proposed glutamate-assisted mechanism comes from experimental and computational studies on nucleotide triphosphate (NTP) hydrolysis in other NTPases,^34,63,64,66,67^ which, like AAA+ proteins, share the Walker B motif and a highly conserved glutamate in this region.

### 3.2 Orientation of the Catalytic Water by Sensor 1

For the glutamate-assisted mechanism, we identify two reaction channels. Channel A, where a water molecule attacks that is stabilized and oriented by hydrogen bonds to Sensor 1 Asn348, and channel B, which involves a different water molecule. In Figure 5, both water molecules are shown in blue and orange, respectively, together with the key interatomic distances to P_γ_ and Glu305. The channel A water molecule (blue) is further away from P_γ_ than the channel B water, but already well positioned for nucleophilic attack, resulting in a smooth reaction energy profile (blue curve in Figure 4). Additionally, the channel A water molecule is stabilized by a strong H-bond formed with the amide of the peptide bond between Asn348 and Thr347 and a weaker H-bond, as well (see also Figure S11). This buried water molecule is also part of the crystal structure, located within 3 Å from the H-donor N of the asparagine and 5.3 Å away from the P_γ_ atom (see Figures S3, S10, and S11).

**Figure 5:**
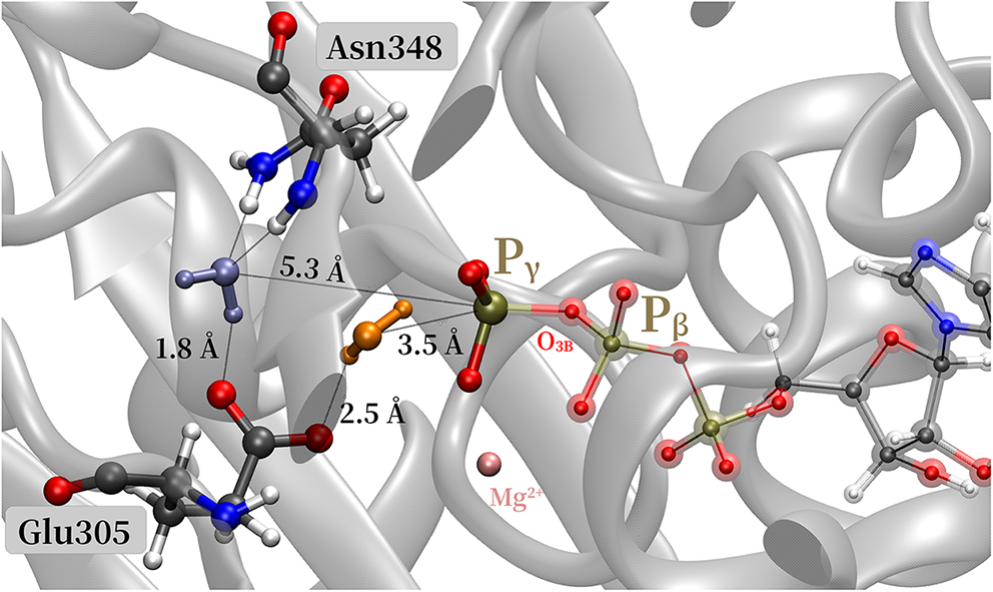
Binding pocket: two water molecules close to Glu305 and the P_γ_ atom. The water molecule marked by violet blue is oriented and stabilized by hydrogen bonds with the Sensor 1 Asn348 (channel A). The water molecule marked by orange is the closest to the *P*_γ_ atom (channel B).

The channel B water molecule (orange) is closer to P_γ_, but it is not well positioned for hydrogen transfer to Glu305 (see also Figures S9–S11 in the SI). Hence, the corresponding MEP of Figure 4 (orange) shows two reaction barriers, where the first corresponds to the reorientation of the water molecule to a hydrolysis-competent state, where the attacking angle is more optimal. Active site configurations for the tested water positions and the resulting adiabatic mapping pathways are shown in Section S2 of the SI.

Figure 6 shows the minimum energy pathways as obtained from NEB calculations together with key interatomic distances (O_Wat_-P_γ_ in red, O_3B_-P_γ_ in green, and proton distance to Glu305 in blue). The first nine images of the channel B NEB path capture the reorientation of the water molecule to a position where it is closer to the proton acceptor, while the O_Wat_-P_γ_ distance does not change. At the same time, the H^+^ approaches Glu305 to 1.8 Å. This distance of the O_Glu305_-H_Wat_ does not change for channel A as the preoriented water molecule only needs to get closer to the P_γ_. Another important structural characteristic that distinguishes the two channels is the attack angle (Figure S9) that needs to reach a nearly collinear state before hydrolysis occurs. After reorientation of the channel B water, for both channels, the O_3B_-P_γ_ bond cleavage (green) and O_Wat_-P_γ_ bond formation (red) occur concertedly in an S_N_2-like reaction mechanism and with a similar reaction energy barrier.

**Figure 6:**
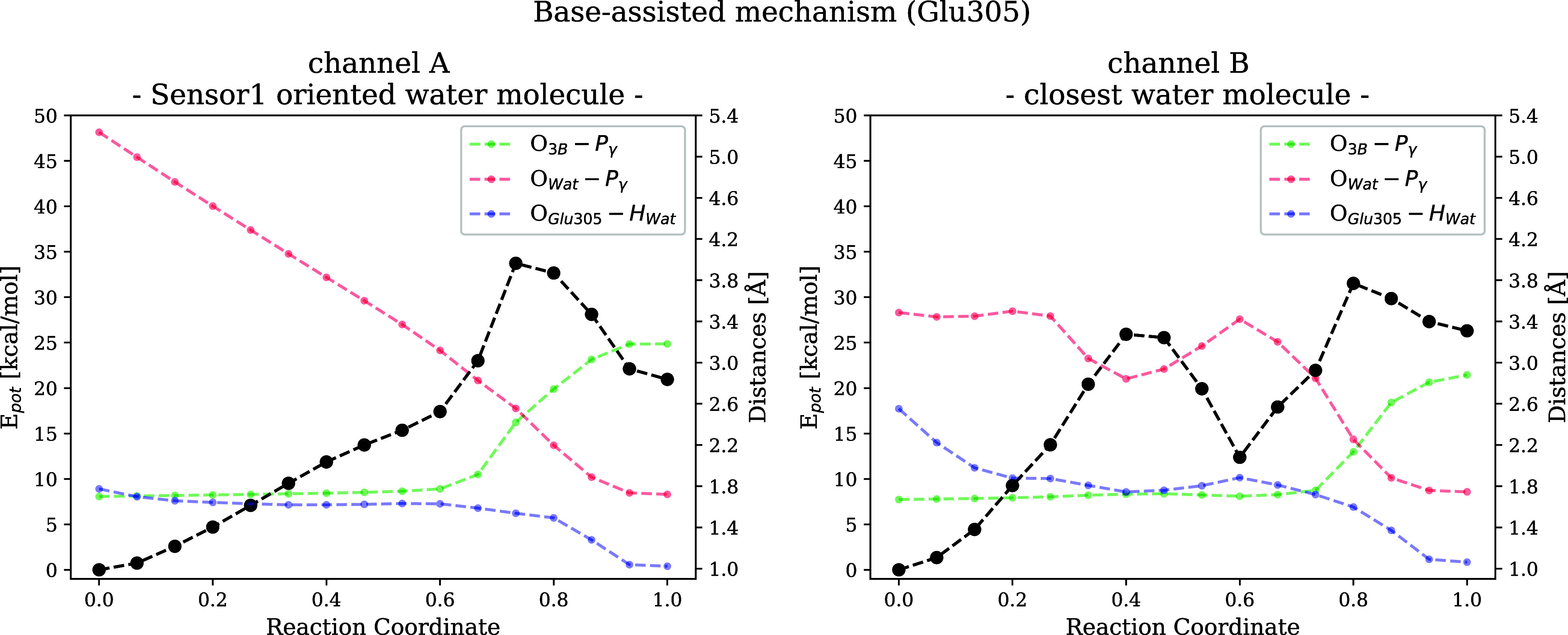
MEPs for channel A and channel B reactions are shown in black. Colored lines denote key interatomic distances for the base-assisted mechanism, with the corresponding axis given on the right.

Therefore, we conclude that the reaction requires a water molecule that is well positioned for the nucleophilic attack as well as hydrogen-bonded to the proton-accepting Glu305. The Sensor 1 Asn348 provides perfectly preoriented water molecules, whereas the nucleophilic attack of other waters involves an additional reorientation step, reaching an activated intermediate configuration. In line with our observation of the Asn348 residue’s role in p97, a recent QM/MM study on helicase-catalyzed ATP hydrolysis^64^ similarly reported that a hydrogen bond involving the backbone of a glycine residue at the active site helps to orient the nucleophilic water molecule for the attack.

### 3.3 QM/MM-MD Conformational Sampling Lowers the Activation Barrier

In the next step, the optimized MEP serves as PCV for free energy simulations. Because of the high cost of these simulations, we select only the most promising MEP, the one corresponding to the attack of the Sensor 1-oriented water molecule in a base-assisted (Glu305) mechanism. MD simulations are initiated from every image of the optimized NEB path and a reaction free energy profile is computed from QM/MM-MD simulations using the path Well-Tempered Metadynamics extended-system Adaptive Biasing Force (WTM-eABF) algorithm as implemented in our Adaptive-Sampling package.^60^

In Figure 8, the resulting PMF (right), as well as trajectories (left) and histograms (middle) of the PCV for the two simulations that start from both minima, are shown. The trajectories of 14 additional simulations are shown in Section S10 of the SI, reaching a total sampling time of 2 ns. A PMF from the data of all combined simulations is shown in gray on the right side of Figure 8. The trajectories (left) show how the system is reversibly driven from one basin to another. The first transition in the forward direction occurs after 23–30 ps as the system has to overcome a high energy barrier in this direction, while the first transition in the backward direction takes place in less than 25 ps. The histograms of the PCV show a sufficiently uniform distribution over the sampled 200 ps, during which four full transitions occur between the educt and the intermediate structures. This suggests that the employed WTM-eABF algorithm effectively enhances sampling. It drives the system across the free energy landscape, allowing even exploration of the reactant, transition state, and product regions. For further details, see Figures S24–S25, which illustrate the evolution of key interatomic distances during sampling. Efficient enhancement of the QM/MM-MD sampling was essential for two reasons: the large size of the QM region (Figure 7) and the extended simulation time. The QM region consists of 164 atoms, selected around the reaction center defined by the attacking water molecule, the P_γ_ atom, and the catalytic base E305, as illustrated in Figure S16 of the Supporting Information.

**Figure 7:**
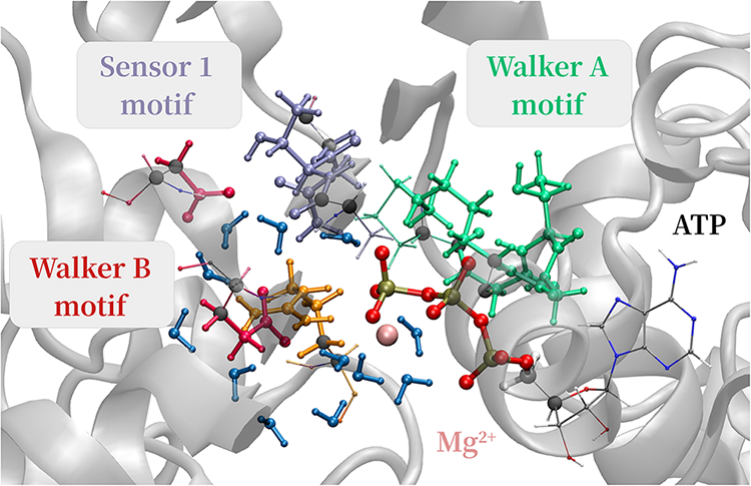
First step: the QM region consist of 164 atoms: the phosphate backbone of the ATP molecule, the 12 closest water molecules (blue), Glu305 and Asp307 from the Walker B motif (red), Ala346, Thr347, and Asn348 from the Sensor 1 motif (purple), Gly248, Thr249, Gly250, and Lys251 from the Walker A motif (green), as well as Arg359 from the adjacent protein subunit (orange). Gray spheres indicate carbon atoms at the QM/MM boundaries, where hydrogen link atoms were introduced; only nonpolar C–C bonds were cut to define the QM region.

**Figure 8:**
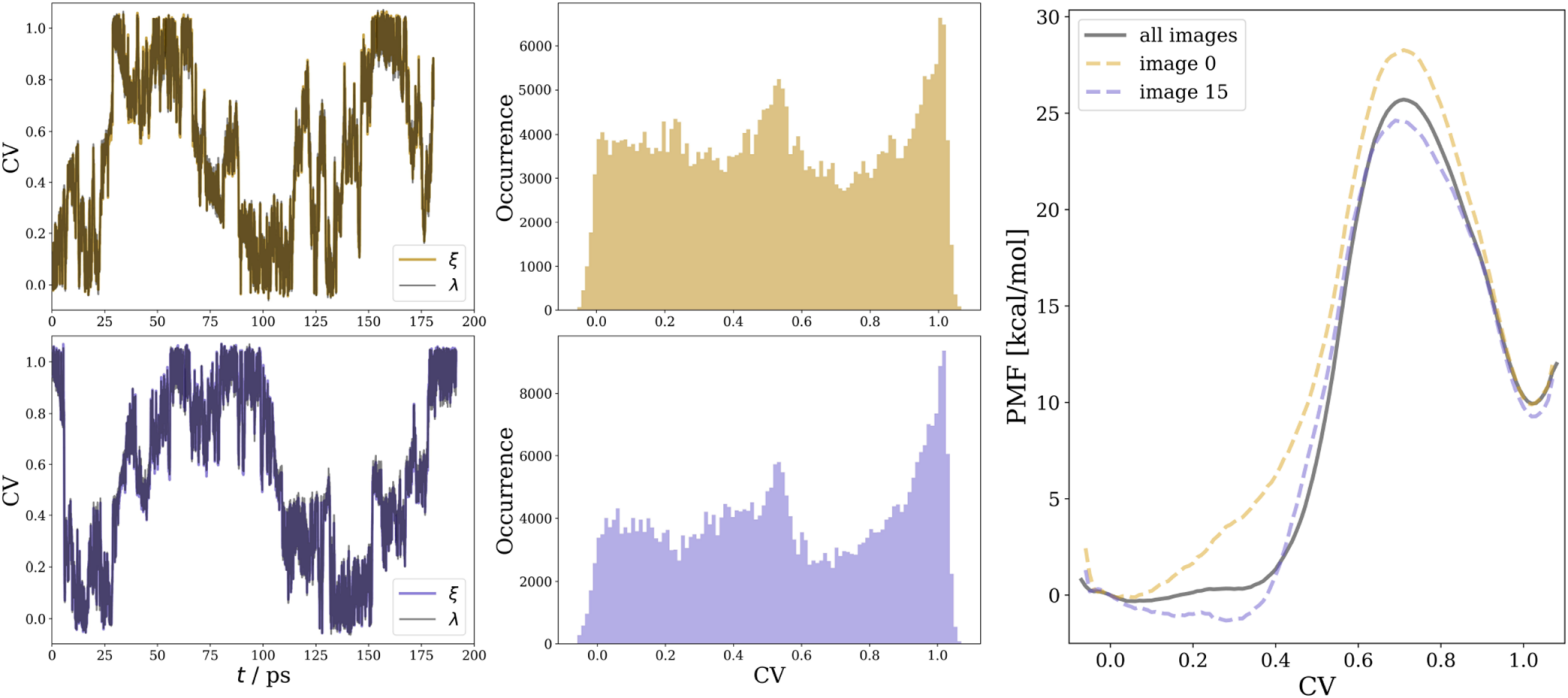
Left: Trajectories and histograms of the PCVs started from the educt (gold) and intermediate structure (purple). Right: PMF profiles were computed for QM/MM-MD trajectories initiated from the respective NEB images. The gray free energy profile was computed from the cumulative data of all 16 trajectories started from different NEB images. The PCV represents the hydrolysis reaction progress along the minimum free energy path, i.e., “0” corresponds to ATP and “1” to the ADP + HPO_4_^2–^ intermediate.

The free energy barrier (25 kcal/mol) obtained from PMF is significantly lower than the static NEB result (35 kcal/mol). Furthermore, the intermediate is also strongly stabilized, as evidenced by the local minimum corresponding to the ADP + HPO_4_^2–^ state at about 10 kcal/mol in contrast to the 20 kcal/mol located on the MEP (see Figure 6).

From the experimentally measured reaction rate constant for ATP hydrolysis in p97,^8,20^ we estimate the activation free energy using the Eyrings equation $k=\frac{\kappa k_{B}T}{h}\exp\left[-\frac{\Delta G^{‡}}{\mathrm{RT}}\right]$ , where *κ* is assumed to be 1, *k*_B_ is the Boltzmann constant, *T* is the temperature used in the experiment, *h* is Planck’s constant, and Δ*G* ^‡^ is the activation free energy. According to this, a free energy barrier of at least 19.67 kcal/mol corresponds to the experimentally measured *k*_hydrolysis_ = 20 min^–1^ (50 °C) rate constant,^20^ which is in reasonable agreement with the obtained results. It is important to note that while a 2–5 kcal/mol error can be standard for hybrid DFT applications,^68^ the slight overestimation of the barrier in our case can be attributed to the confinement of the path CV. When including the harmonic confinement potential into the MBAR analysis, the free energy barrier is reduced and closer to the experimentally derived value (see Figure S22 and the discussion in Section S7).

### 3.4 Forming the Product under the Rearrangement of the Mg^2+^ Coordination Shell

After the first step, we reach a high-energy intermediate at the active site, which corresponds to ADP, HPO_4_^2–^, and the protonated Glu305. The last step consists of a H^+^ transfer from the Glu305 to the O atom of the P_γ_ atom, forming H_2_PO_4_^–^, the inorganic phosphate (P_i_). An optimized NEB path together with key interatomic distances is shown in Figure 9. In black, the MEP for this reaction is shown, which has a low activation barrier of 7 kcal/mol and is strongly exothermic by about 19 kcal/mol. The reaction is characterized by two structural rearrangements: first, the proton from the catalytic base is transferred to the O_2G_ oxygen atom of P_γ_ (gray line of Figure 9), which coordinates Mg^2+^ (see Figure 10), likely due to steric proximity or the high electron density of this oxygen atom. This assumption is supported by a ^19^F NMR study on GTP hydrolysis,^69^ showing that the oxygen atom coordinated to Mg^2+^ has the highest electron density among the oxygens of P_γ_ and is the proton acceptor.^32^ Second, the proton transfer step also requires a rearrangement of the divalent ion coordination shell, as shown by the purple and blue lines that denote the evolution of the Mg^2+^-O_3B_ and Mg^2+^-O_Thr252_ distances.

**Figure 9:**
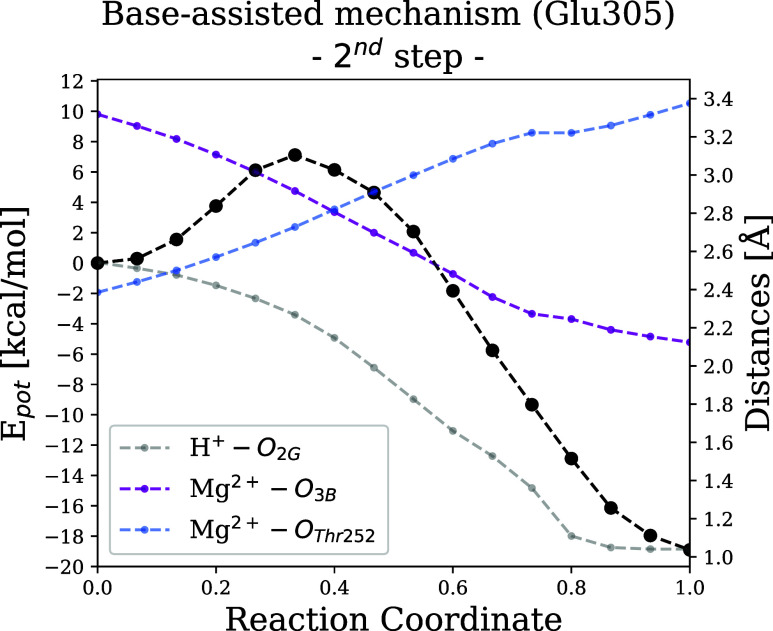
MEP of the second reaction step is shown in black (left axis) and key interatomic distances in gray, purple, and blue (right axis).

**Figure 10:**
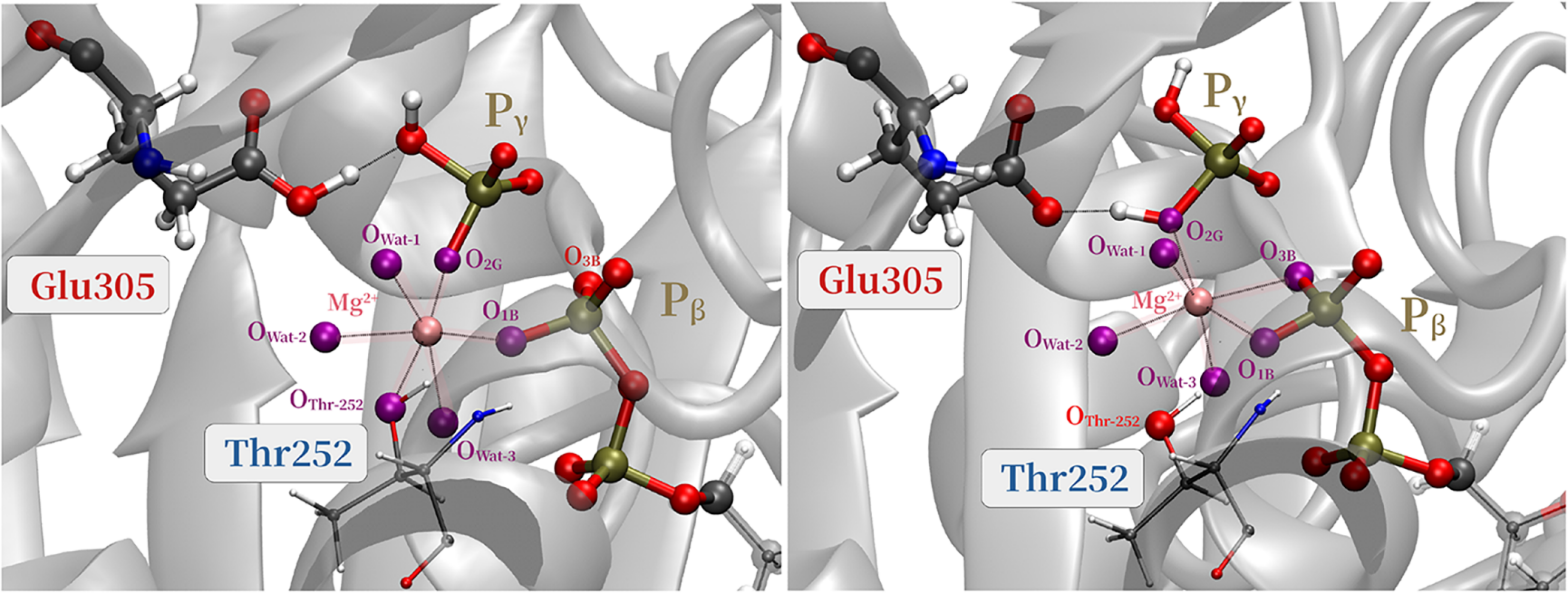
Second step: Mg^2+^ coordination shell in the intermediate (left) and product (right) states.

As shown in Figure 10, both in the intermediate and product states, Mg^2+^ is tightly coordinated by six ligands, forming an octahedral geometry. A key event in the second step is the reorganization of the Mg^2+^ coordination shell. In both the educt and intermediate states, Thr252—a highly conserved residue across many P-loop NTPases^11^—strongly coordinates to Mg^2+^. However, upon H_2_PO_4_^–^ formation, the threonine leaves the ion’s coordination shell and O_3B_ becomes part of it. This observation aligns with the high-resolution cryo-EM structure,^8^ which captures the ADP·P_i_ state of human ATPase p97 just before the release of the inorganic phosphate from the binding site. The cryo-EM density of this study also indicates that compared to the ATP-bound state in the product, the Mg^2+^ ion has already dissociated from the threonine. The rearrangement in the coordination shell leads to a 3-fold connection between Mg^2+^, ADP, and P_i_ which remains stable for 10 ps in an unbiased QM/MM-MD simulation of the product state (see Section S8 of the SI), after which a water molecule enters the coordination shell.

As shown in Figure 11, in addition to the coordination shell, a strong H-bonding network stabilizes the product state. The Walker A residue Lys251—the immediate neighbor of Thr252—acts as a tridentate, contacts O_3G_ and O_2B_, and thus bridges ADP and P_i_. The inorganic phosphate is further stabilized by forming H-bonds with the H^+^ donor Glu305 and Arg359, which are stronger than those of the educt state (see Section 8 of the SI).

**Figure 11:**
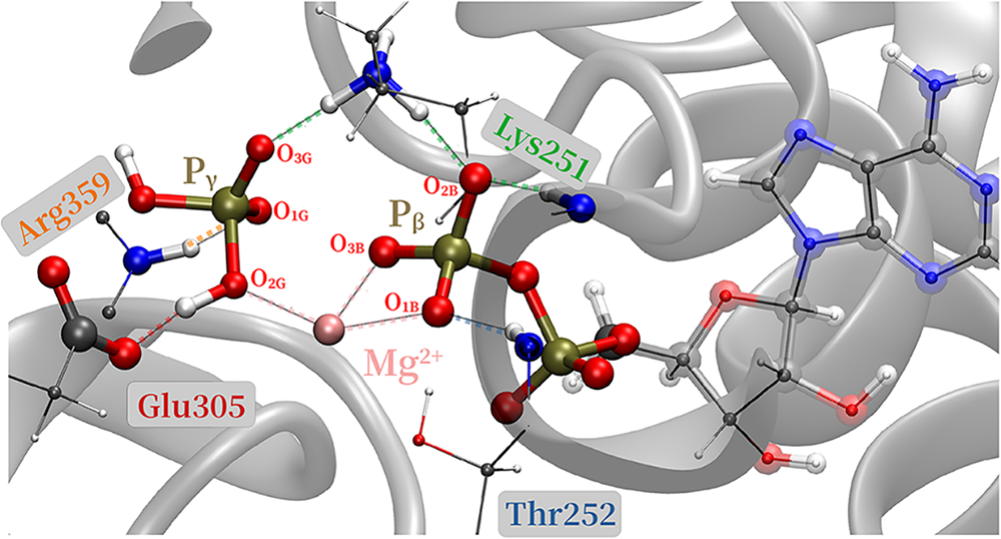
Key protein residues stabilize the product state.

We conclude that the minimum energy pathway of the proton transfer step leads to a product state, where the strongest electrostatic interactions between the substrate and protein residues occur with the Mg^2+^ ion and the Lys251 side chain, both of which were identified as key stabilizers of the post-hydrolysis ADP·P_i_ state.^8^ The unbinding of the inorganic phosphate has a high kinetic barrier^20^ and will be anticipated by a rearrangement in the Mg^2+^ coordination shell, where the inorganic phosphate detaches and the vacant places are occupied by neighboring water molecules. However, studying the detachment process of the inorganic phosphate is outside the scope of this paper.

### 3.5 Comparison to Solid-State NMR Measurements

In our previous work,^35^ we have discussed computed NMR shifts as ensemble properties predicted from microseconds-long MM-MD trajectories of the educt and the product state without studying the reaction mechanism of ATP hydrolysis. The product state in this case featured ADP bound to p97 without the cleaved inorganic phosphate, which had already detached from the binding site. Here, we have observed that pre- and post-hydrolysis chemical shifts of the nucleoside differ the most for the P_β_ nucleus.^35^ The chemical shift computed for this nucleus undergoes a drastic downfield shift of more than 12 ppm, showing a good agreement with experimental NMR measurements.^20^ After the reaction mechanism of ATP hydrolysis is studied, NMR calculations are performed using a QM/MM DFT framework, allowing the computation of NMR chemical shifts along the reaction pathway as one transitions from the educt to the product structure. Compared to NMR measurements, which capture averaged chemical shifts over the acquisition time, this enables direct observation of the influence of cleavage of the phosphate bond in the first step, as well as proton transfer and Mg^2+^ coordination in the second step on chemical shifts.

The results of this analysis are shown in Figure 12 in combination with key interatomic distances. There are more factors that contribute to these changes in the chemical shifts; here, however, we restrict our discussion to the key events that are observed during the first and second reaction steps. For the P_α_ nucleus, which is not directly involved in the reaction, chemical shifts barely change during the two reaction steps. In contrast, for the P_β_ nucleus, a 16 ppm downfield shift is observed upon hydrolysis, showing an excellent agreement with the experimentally observed difference between the shift measured before (ATP: −20 ppm) and after hydrolysis (ADP: −4 ppm) (see Figure 2 of the experimental NMR study^20^). The contribution of the second reaction step to the overall downfield shift of the P_β_ nucleus is significant (see the right panel of Figure 12). This is not surprising as we have already observed how the Mg^2+^ coordination sphere in the immediate proximity of the P_β_ nucleus changes as the reaction progresses toward the product state (see Figure 10). The O_3B_ atom enters the coordination shell, while Thr252 leaves it, as captured by the MEP.

**Figure 12:**
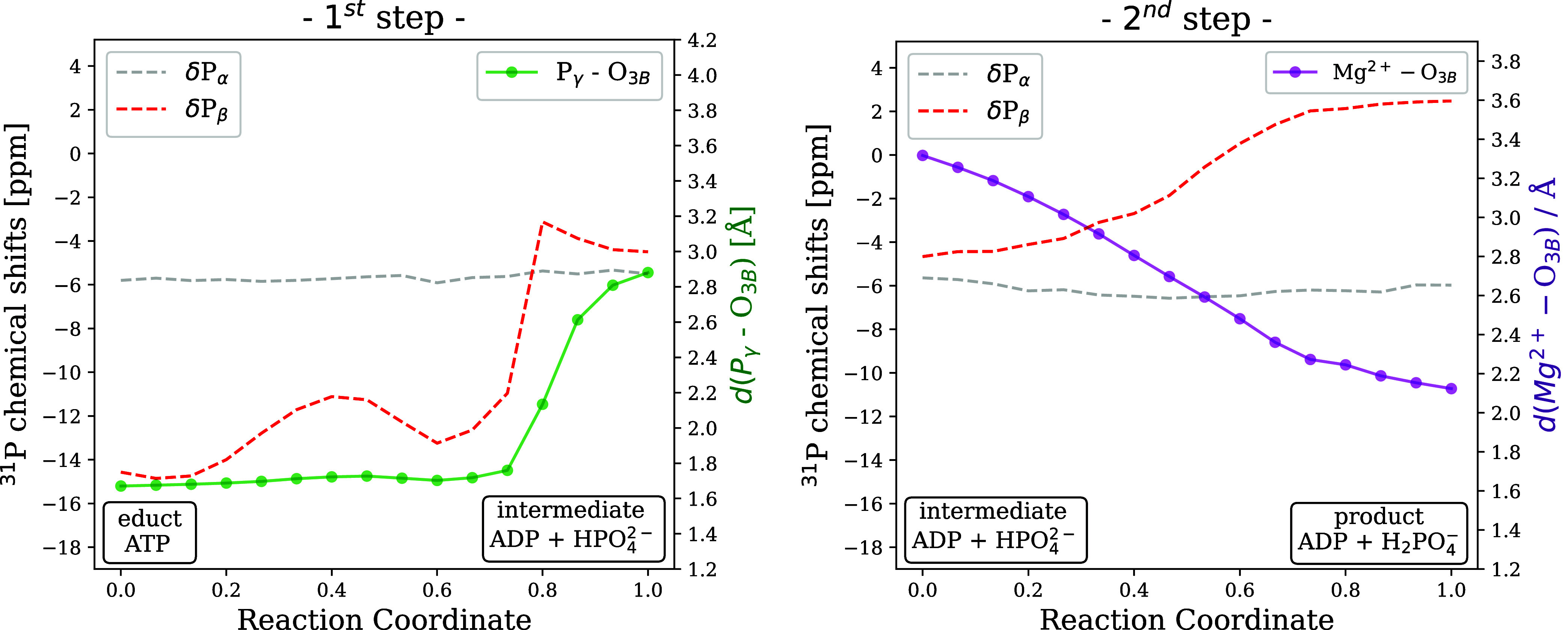
Monitoring ^31^P NMR chemical shifts along the two-step reaction progress. The dashed lines show the chemical shifts of the P_α_ (gray) and the P_β_ nucleus (red). Continuous lines with points represent the measured interatomic distances during the 1^st^ and 2^nd^ reaction steps (P_γ_-O_3*B*_ in green and Mg^2+^-O_3B_ in purple).

Further, as the scissile P_γ_-O_3B_ bond elongates (green line in Figure 12), the P_β_ shift immediately moves toward the downfield region, eventually reaching −5 ppm, such that the intermediate state already has a drastically changed P_β_ shift. The wild-type ADP-bound spectra show a downfield-shifted P_β_ signal, and based on the computed NMR chemical shifts along the reaction progress, we conclude that this change happens already upon hydrolysis, rather than only during the inorganic phosphate (P_i_) release. Therefore, the experimental observation of a P_β_ shift in the upfield region near −15 ppm in the post-hydrolysis ADP.P_i_ state^20^ remains elusive, likely due to the use of the E305Q mutation needed for the ADP.P_i_ NMR measurements, which could lead to a mixture of ATP-bound and hydrolyzed species.

## 4 Conclusions

In this work, we applied a hybrid QM/MM approach that combines minimum energy pathway optimizations and enhanced sampling methods to provide mechanistic insights into the protein-mediated ATP hydrolysis catalyzed by p97. Our findings clarify the role of various amino acids at the binding site:

- Glu305 from the Walker B motif, a highly conserved feature in many AAA+ proteins, catalyzes the reaction by accepting a proton from the catalytic water, which is later transferred back to the inorganic phosphate to form the final product.
- The Asn348 residue of the Sensor 1 motif, also present in all AAA+ proteins, orients the attacking water molecule. A crystallographic water molecule is positioned near a strong proton acceptor (Glu305), stabilized by hydrogen bonds, and aligned almost collinearly with the P–O bond that is about to be cleaved.
- The coordination shell of the Mg^2+^ ion stabilizes the transition state and product together with positively charged protein residues such as Lys251 of the Walker A motif and Arg359 of the Walker B motif. To form the product, the Walker A Thr252 leaves the Mg^2+^ coordination shell in favor of the O_3B_ of P_β_.

Furthermore, we show that a single water molecule can hydrolyze ATP and that phosphate bond breaking and bond formation occur concertedly in a single reaction step. For studying the rate-limiting step of hydrolysis, we apply extensive sampling and obtain an activation free energy barrier that is in reasonable agreement with the experimental catalytic turnover rates. Starting from the pre-hydrolysis crystal structure, our computational exploration of the reaction mechanism finally leads to a conformation that is consistent with the cryo-EM structure of the post-hydrolysis protein state. Finally, we complement our mechanistic insights by exploring how ^31^P NMR chemical shifts evolve along the proposed ATP hydrolysis pathway, linking their drastic changes, which are also observed in experimental NMR studies,^20^ to key structural rearrangements. Overall, our contribution provides a full picture of the chemical step of ATP hydrolysis catalyzed by p97.

## Supplementary Material



## Notes

Computational details about the adiabatic mappings and NEB minimum energy pathway calculations; method and basis set benchmark calculations for the minimum energy pathways as well as a QM region size convergence study; details about the QM/MM-MD enhanced sampling simulations, MBAR reweighting, uncertainties associated with the computed free energy profiles, details about the DFT NMR calculations; and illustrations of the H-bonds between the substrate and key active site residues in the educt and product states (PDF)
