# Supplementary material for: Molecular Mechanism of ATP Hydrolysis Catalyzed by p97: A QM/MM Study
Source: J Chem Theory Comput. 2025 Sep 19;21(19):9459–69. doi: 10.1021/acs.jctc.5c00928 (PMC12529919; doi:10.1021/acs.jctc.5c00928)
Supplement: Supplementary file 1 [file ct5c00928_si_001.pdf]

Supplementary Material: On the Molecular Mechanism of ATP Hydrolysis Catalyzed by p97: a QM/MM Study

Judit Katalin Szántó,[1] Andreas Hulm,[1] Christian Ochsenfeld*,[1,2]

[1]Chair of Theoretical Chemistry, Department of Chemistry,
University of Munich (LMU), Butenandtstr. 7, D-81377 München, Germany,
[2]Max Planck Institute for Solid State Research,
Heisenbergstr. 1, D-70569 Stuttgart, Germany
*E-Mail: christian.ochsenfeld@uni-muenchen.de

Contents

1 QM/MM calculations S3

2 Structure optimizations and adiabatic mappings S4

3 Finding minimum energy pathways using the nudged elastic band method S9

4 Benchmark calculations - the influence of different DFT functionals and basis sets S12

5 Benchmark calculations - the influence of the cutoff of electrostatic interactions with the MM subsystem S15

6 Benchmark calculations - QM region size S16

7 Enhanced sampling using the WTM-eABF method S21

8 The educt and the product state - H-bond networks at the binding site S25

9 DFT NMR calculations S28

10 Enhanced sampling using the WTM-eABF method - trajectories and histograms for the first reaction step S29

11 Evaluation of the PMF profile uncertainties S35

References S37

1 QM/MM calculations

QM/MM simulations were performed in our in-house program package FermiONs++[1–3]. Unless otherwise noted, for the QM part Grimme's PBEh-3c DFT functional was used [4]. Significant speed-ups were achieved using the sn-LinK method [5–7] and the RI-J approximation [8] for fast evaluation of exact exchange and Coulomb energy terms, respectively. An automatic workflow was employed to place H-atoms as links between the MM and QM region. These link atoms were introduced between C_β and C_α atoms if single amino acids were selected into the QM region (Figure S1 a) and between C_α and C atoms in case of amino acid sequences (Figure S1 b). The QM/MM boundaries were chosen such that peptide bonds along the protein backbone and polar bonds were not cut.

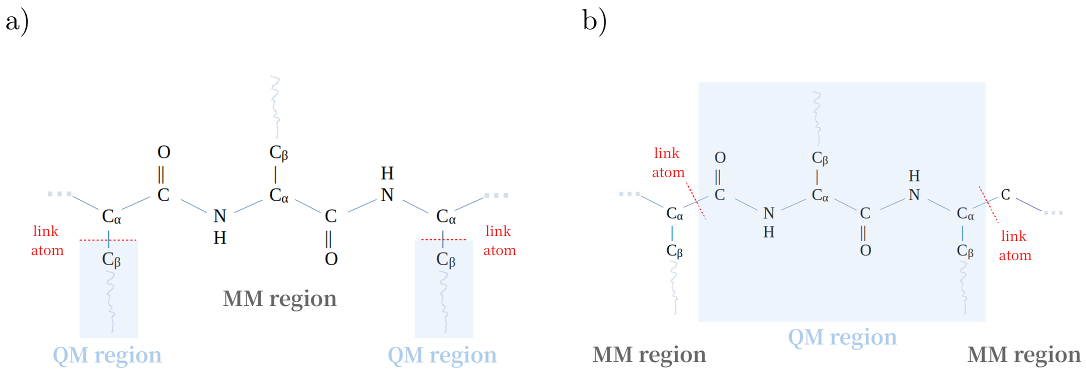

Figure S1: Representation of QM/MM partitioning scheme: a) Bonds between the C_α and C_β atoms were cut when including single amino acids in the QM region and b) Bonds between the C_α and C atoms were cut when including a series of amino acids.

QM/MM calculations were carried out with the equilibrated educt structure, which in contrast to the static X-Ray structure contains amino acids in a catalysis-ready conformation.

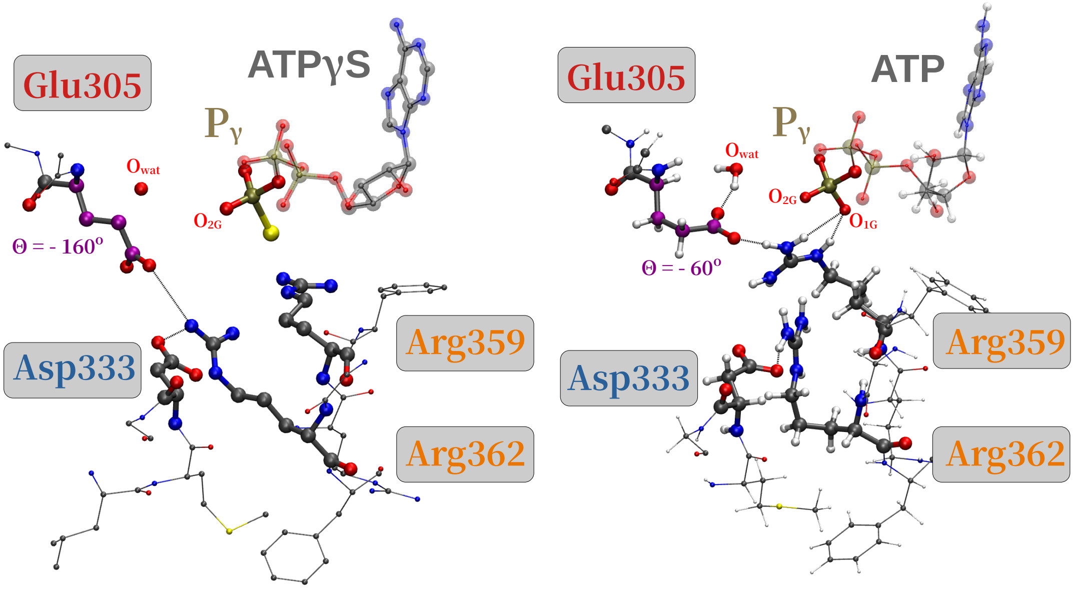

Figure S2: Comparison of the active site conformations in p97. Right: X-ray structure (PDB: 4KO8) with the non-hydrolyzable ATP analog ATPγS. Left: ATP-bound protein state. Key residues involved in nucleotide sensing and catalysis (E305, R359, R362, D333) are shown to illustrate conformational differences.

2 Structure optimizations and adiabatic mappings

For the adiabatic mappings (AMs), snapshots were extracted from the MM-MD simulations of the ATP-bound state of p97 [9, 10], followed by QM/MM structure optimizations. The DL-Find library [11] implemented in PyChemShell [12] was used for structure optimizations connected to the Python-interface of FermiONs++. Convergence criteria for DFT QM/MM structure optimizations were set as follows:

The optimized structures were used as starting points for the adiabatic mapping pathways, where reactants were stepwise optimized while pulling along a predefined collective variable (CV) using harmonic restraints with a force constant of 1 kJ/molÅ^2. The used collective variable is the $d(O_{wat} - P_\gamma)$ distance (see Fig. S7 and Fig. S6) or the linear combination of the bond formation ($d(O_{wat} - P_\gamma)$) and bond cleavage ($d(P_\gamma - O_{3B})$) (see Fig. S5), as implemented in the *colvars* module of the adaptive-sampling python package[13]. The proton transfer from the nucleophilic water is not explicitly biased,

Table S1: Convergence criteria for structure optimization of ATP in p97

Criteria	Threshold
Energy	$4 \times 10^{-4}\,E_h$
RMS gradient	$1 \times 10^{-3}\,E_h$
Max. gradient	$8 \times 10^{-3}\,E_h$
RMS step	$5 \times 10^{-3}\,E_h$
Max. step	$5 \times 10^{-1}\,E_h$

allowing the proton to migrate freely without being constrained by the reaction coordinate. Constrained optimizations were performed along the chosen reaction coordinate. In each step, the CV value is, changed and then fixed, while the system was minimized. All residues in a radius of 10 Å around the ATP molecule were relaxed. The QM region contained 126 atoms from amino acids which were found within 3.5 Angstroms around the P_γ, the two O atoms in the GluE305 side group and the attacking water molecule: Gly248, Thr249, Gly250, Lys251, Glu305, Asn348, Arg359, the Mg^{2+} ion at the binding site, and the closest water molecules. From the ATP molecule only the phosphate backbone is included in the QM region, the adenosine remains in the MM subsystem.

Within 6 Å around the P_γ atom, the X-Ray structure contains 8 stable water molecules, without the resolved positions of the protons. Therefore, the first question to answer is which water molecule is a good candidate for the nucleophilic attack.

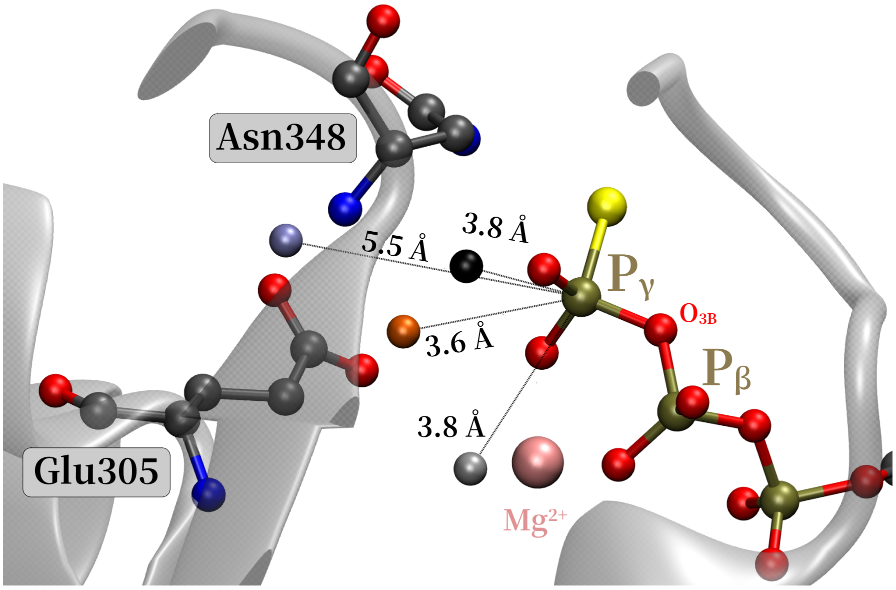

Figure S3: Oxygen atoms of buried water molecules resolved in the X-Ray structure of ATPγS-bound p97 (PDB 4KO8 [14]) and the phosphate backbone of the substrate. The colors of the water molecules correspond to the color coding used in the main manuscript (see Fig. 4-5 in the main text).

In the first approach, we have tested the nucleophilic attack of the closest water molecules to the P_γ and P_β atoms.

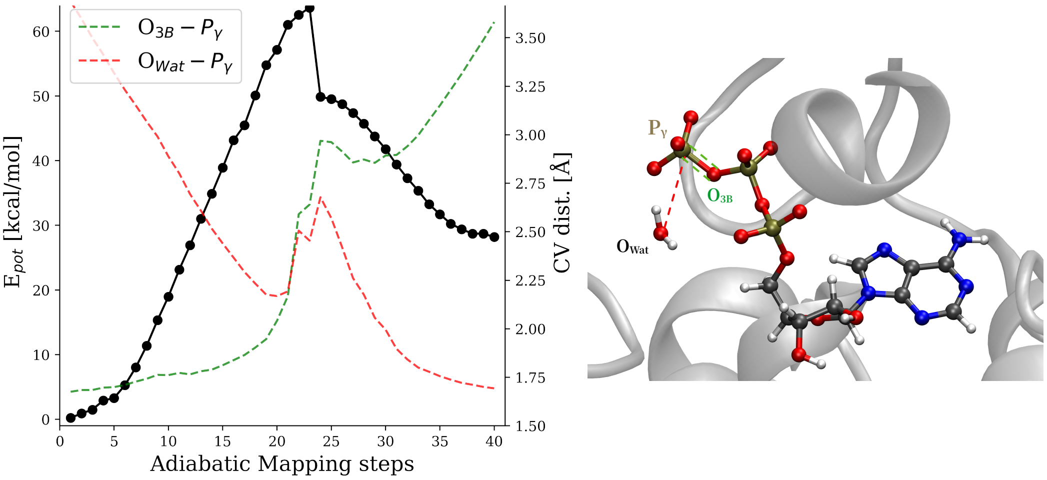

Figure S4: Adiabatic mapping pathway - substrate-assisted mechanism

In the second approach, the two water molecules closest to the Asn348 and the two O atoms of the GluE305 were considered as candidates for the nucleophilic attack. In this simulation, the lytic water molecule is the one closest to the Pg atom and a second, assisting water molecule is the proton donor to the GluE305. A linear combination with decreasing $d(O_{Wat-attack} - P_{\gamma})$ and $d(H_{Wat-attack} - O_{Wat-assist})$ distances was used as collective variable.

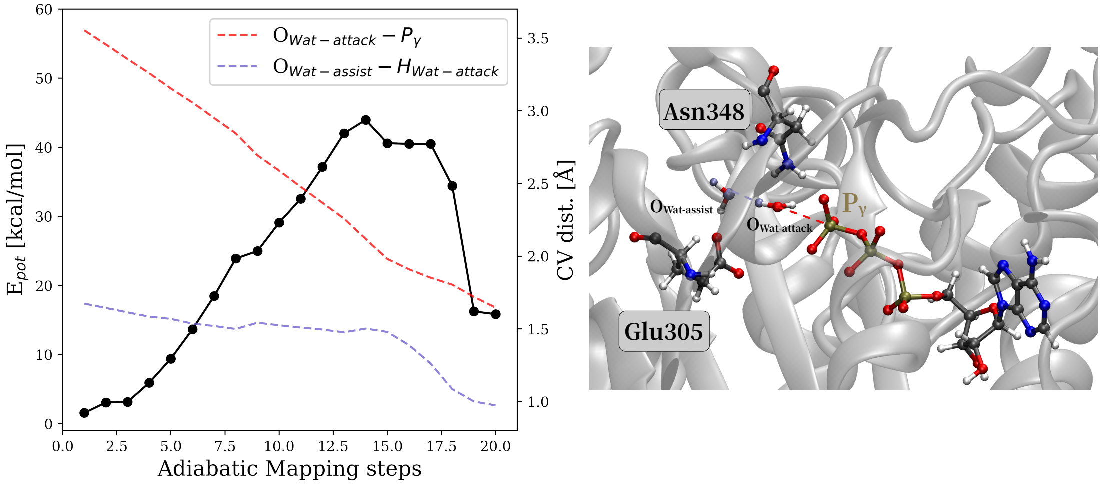

Figure S5: Adiabatic mapping pathway - Base-assisted mechanism (Glu305) - 2 water mechanism

For the single water mechanism the $d(O_{wat} - P_{\gamma})$ distance was used as collective variable.

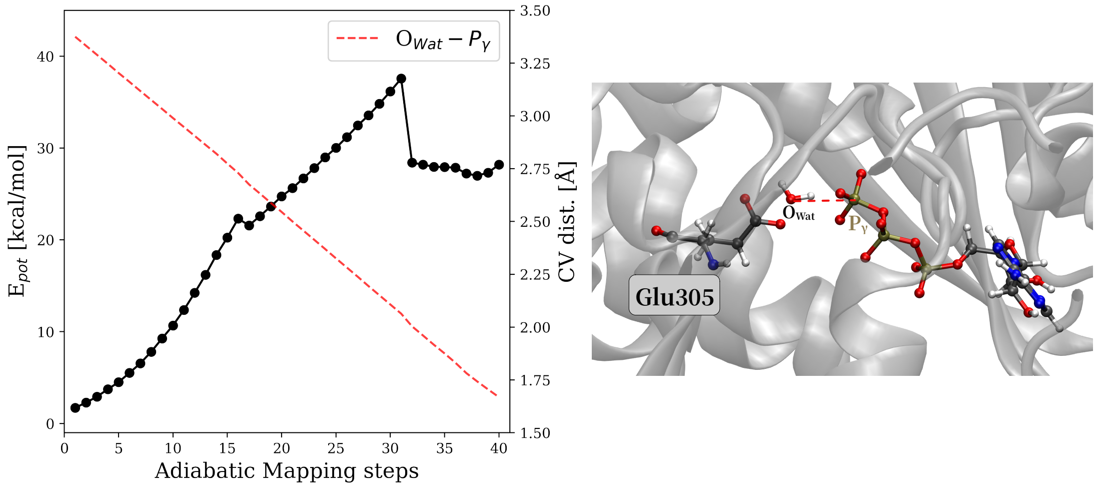

Figure S6: Adiabatic mapping pathway - Base-assisted mechanism (Glu305) - 1 water mechanism (channel B)

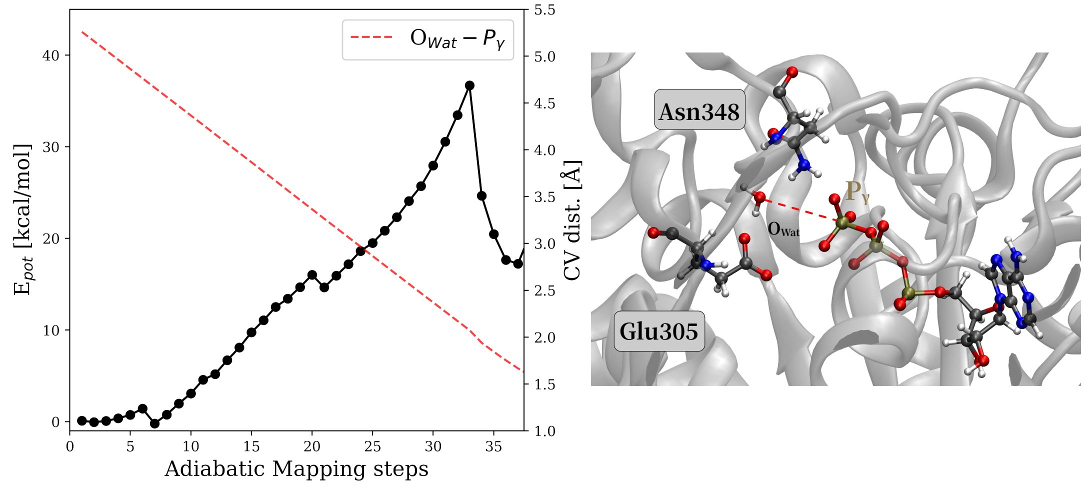

Figure S7: Adiabatic mapping pathway - Base-assisted mechanism (Glu305) - 1 water mechanism (channel A)

After finding adiabatic mapping pathways for the first reaction step, NEB simulations were performed and the glutamate-as-base mechanism was selected as a plausible mechanism. To explore the last step of the reaction and reach the product state, the adiabatic mapping was started from the intermediate reached in the first step. The collective variable for the proton transfer step was defined as the distance between the H^+ and the closest O of the P_γ atom. The CV was decreased by 0.04 Å in each step, and then fixed, while the energy of the system was minimized.

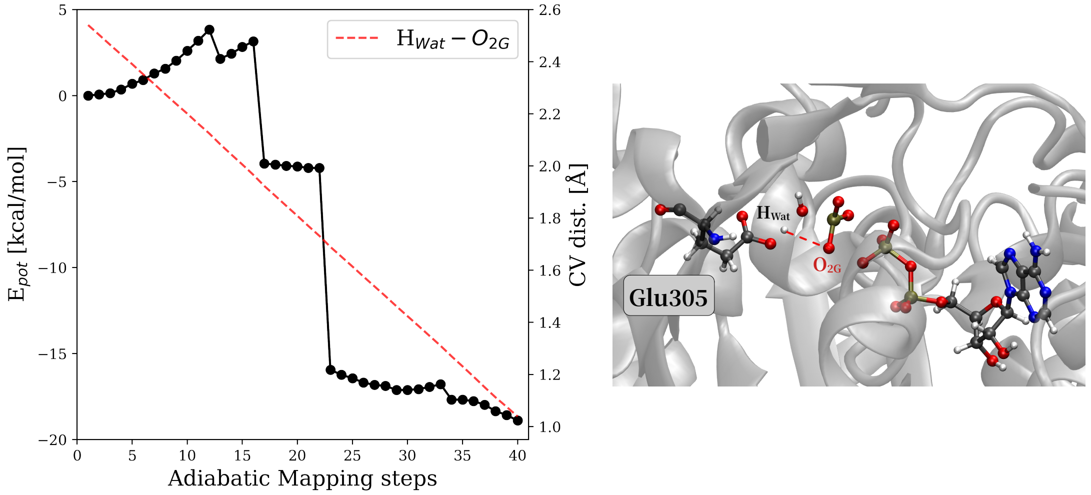

Figure S8: Adiabatic mapping pathway of the second reaction step

There are several key features that influence reactivity, but for this initial exploration,

we used a single distance as the collective variable (CV) or the linear combination of the breaking and forming bond. In all adiabating mapping simulations, the proton migrates freely to GluE305 or to the substrate without being constrained by the CV. AM is highly sensitive to the CV and the initial configuration[15], the pathways we have found using adiabatic mapping show discontinuities, which are very frequent and typical for this approach [16].

3 Finding minimum energy pathways using the nudged elastic band method

NEB simulations were performed using FermiONs++[1–3] together with the FENEB module of the adaptive-sampling package[13]. For each MEP 16 equidistant NEB images were created using linear interpolation between the two optimized endpoints of the adiabatic mapping. 1000 steps of steepest descent optimization were carried out for all NEB images, including the two extreme points corresponding to the reactant and the educt structure. Adjacent images on this path are connected by springs with a force constant k to ensure an equidistant spacing along the pathway. Here, the improved tangent force estimate as described by Henkelmann and Jonsson was applied[17]. The NEB force was used only for selected substrate atoms while the environment was allowed to relax freely. Additionally, in each optimization step the spring force is fully optimized to enforce equidistant spacing of NEB images. This ensured that the resulting MEPs are well suited to serve as path CVs[18] for path WTM-eABF free energy simulations[19–21]. Hence, each image of the optimized minimum energy path was heated and equilibrated for subsequent free energy simulations, confining each MD simulation to its corresponding path node using a harmonic potential with force constant 120 kcal/mol.

For the Glu305 base-assisted mechanisms both reaction channels we observe an almost collinear alignment of the O_{wat}, P_γ and O_{3B} atoms around the TS, before the nucleophilic attack occurs. For channel B the water molecule forms a $\Psi = 135°$ angle in the reactant state, whereas the channel A water molecule forms a $\Psi = 150°$ angle, making it better positioned for the attack.

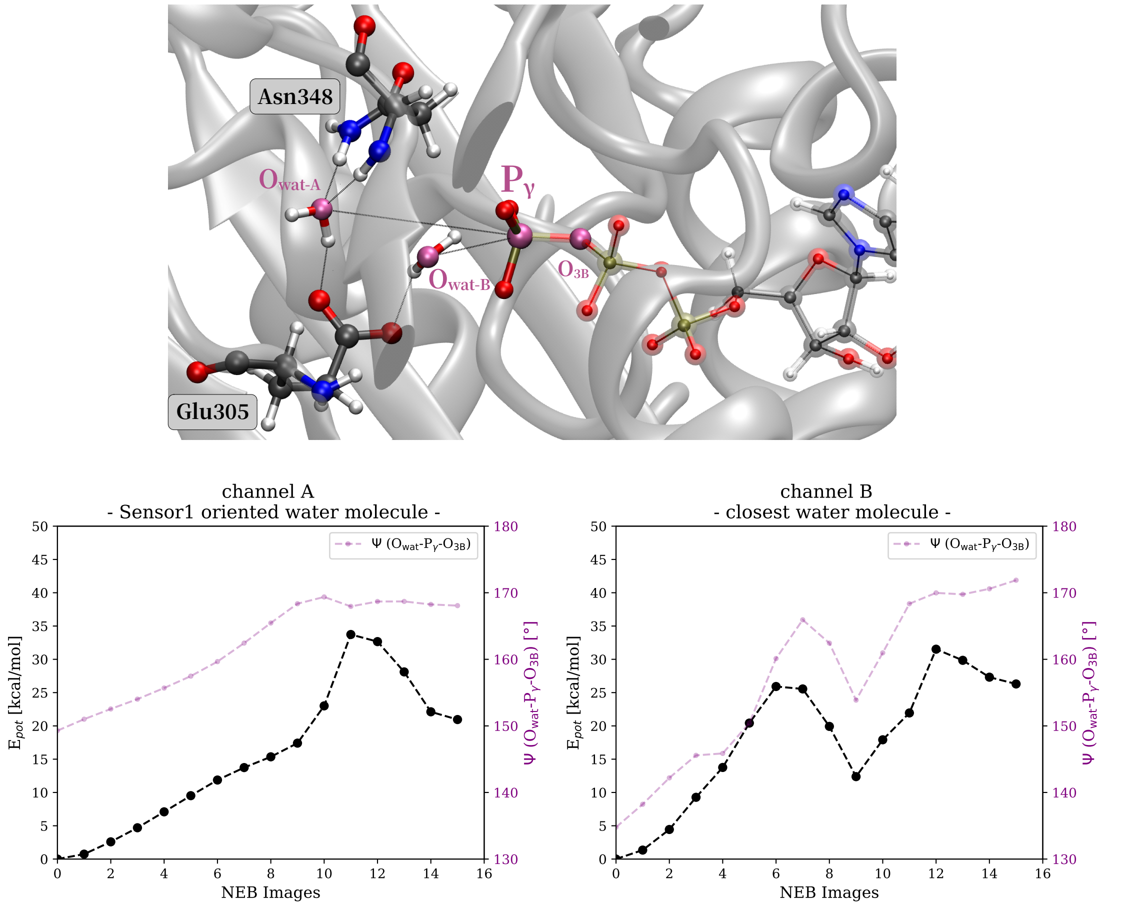

Figure S9: Base-assisted mechanism (Glu). **Top:** the binding site of p97 with ATP, two water molecules corresponding to channel A and B, Sensor 1 Asn348 and the proton acceptor GluE305. Atoms marked with purple have an almost collinear alignment before the nucleophilic attack. **Bottom:** $\Psi(O_{wat} - P_\gamma - O_{3B})$ angle values for images of the channel A and channel B NEB paths.

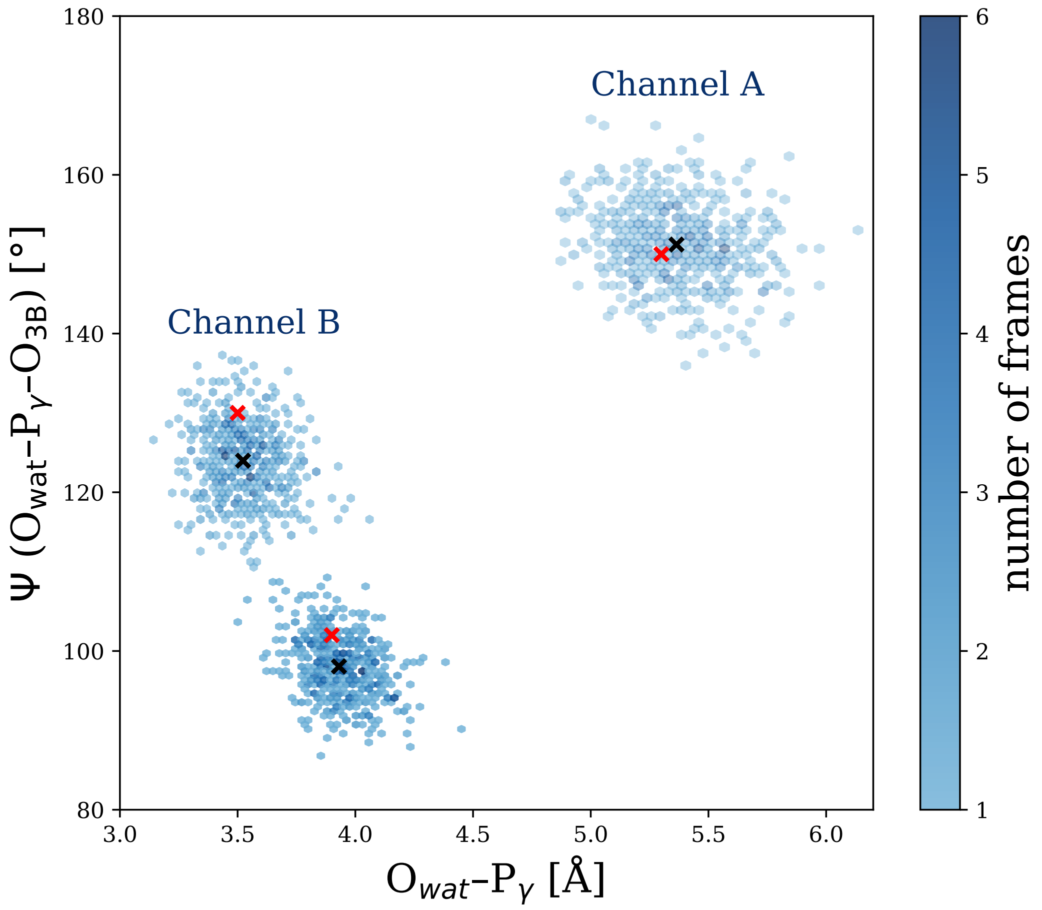

Figure S10: 2D hexbin plot depicting orientations of the closest buried water molecules to the P_γ atom in the active site during the 1-microsecond 1 μs MM-MD trajectory [9]. Black crosses indicate the median values of the $\Psi(O_{\text{wat}} - P_\gamma - O_{3\text{B}})$ angle and $O_{\text{wat}} - P_\gamma$ distance, while red crosses mark the educt structure used in this study. The nucleophilic attack of the water molecule found at $\Psi(O_{\text{wat}} - P_\gamma - O_{3\text{B}}) = 100°$ in the hexbin plot leads to the substrate-assisted mechanism.

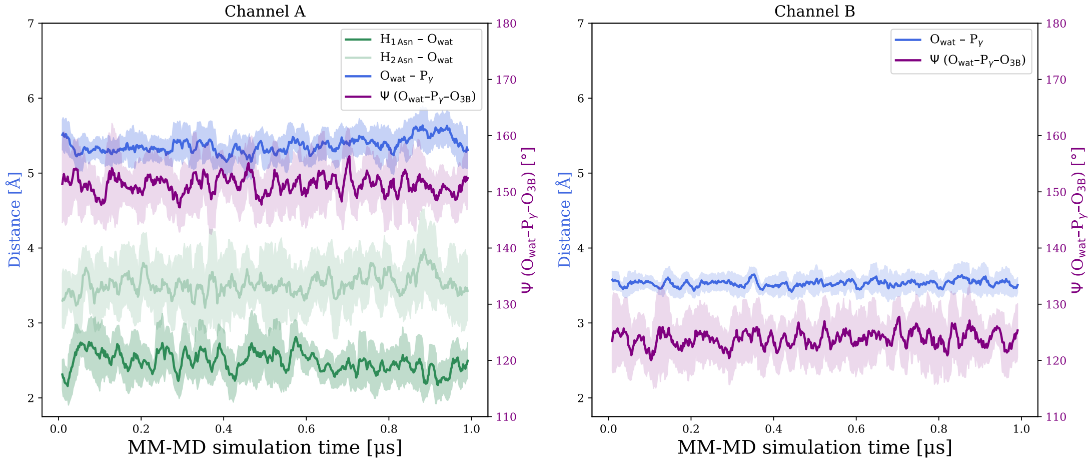

Figure S11: Rolling averages (window size = 10 frames) over the MM-MD trajectory[9] capturing the educt state. Shown are: $O_{wat} - P_\gamma$ distances, H-bonds formed with Asn348 and the $\Psi(O_{wat} - P_\gamma - O_{3B})$ and the attacking angle for the channel A and channel B water molecules. Shaded areas indicate the range of ±1 standard deviation around the rolling average.

4 Benchmark calculations - the influence of different DFT functionals and basis sets

We computed the single point energies of the images from the refined NEB path with various DFT functionals and basis sets using ORCA [22]. We used the following DFT functionals: PBEh-3c, ωB97M-V, B3LYP-D3(BJ) with double-ζ and triple-ζ basis sets. The images of the NEB optimized path obtained with PBEh-3c were used to estimate the electronic energies at different theory levels.

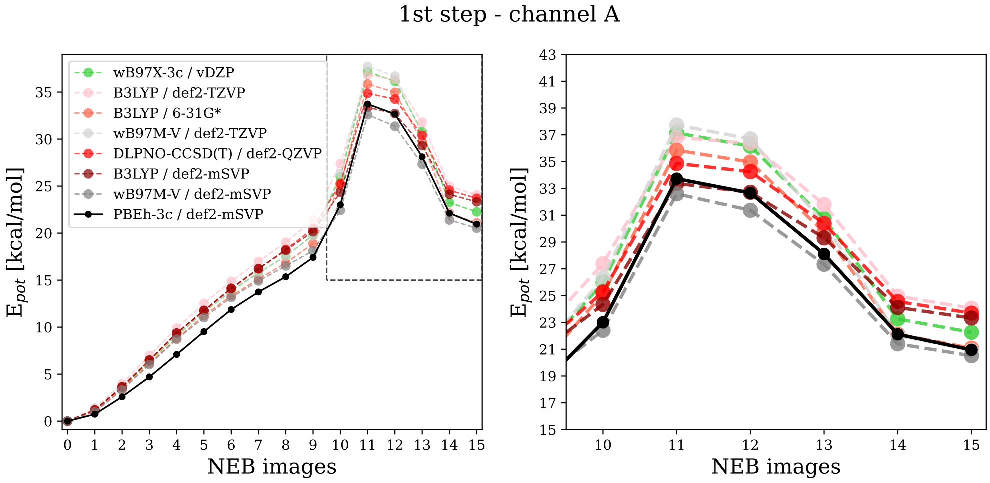

Figure S12: Benchmarking the energetics of the first step, channel A.

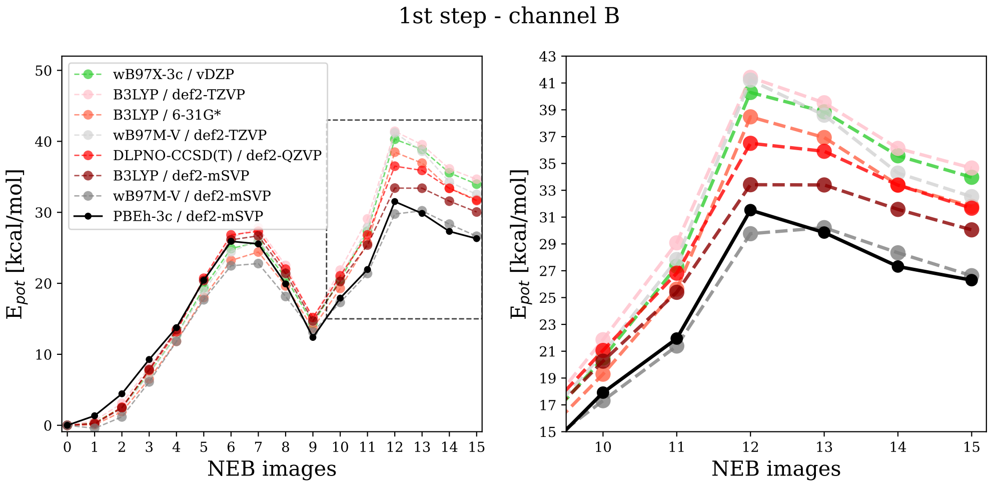

Figure S13: Benchmarking the energetics of the first step, channel B.

For both channels PBEh-3c[4] underestimates the barrier compared to ωB97X-3c[23] by 5 kcal (channel A) and by 10 kcal/mol (channel B). The ωB97X-3c range-separated composite method builds on the ωB97X-V functional, using a molecule-optimized polarized valence double-zeta (vDZP) basis set and a tailored D4 dispersion correction. We find a strong basis set influence. Using triple-ζ basis sets we get very close to the DLPNO-CCSD(T)[24] and ωB97X-3c barrier. We chose the PBEh-3c approach with the double-ζ basis set to enable extensive sampling. Additionally, for all functionals, the activation barrier of channel B is higher than that of channel A, and the intermediate in channel B (6 kcal/mol), representing a shallow minimum, is less stable than the intermediate in channel A (12 kcal/mol).

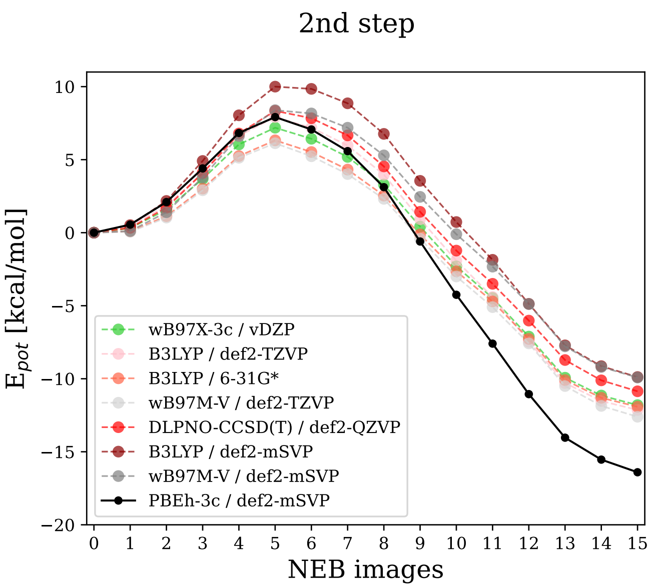

Figure S14: Benchmarking the energetics of the second step.

5 Benchmark calculations - the influence of the cutoff of electrostatic interactions with the MM subsystem

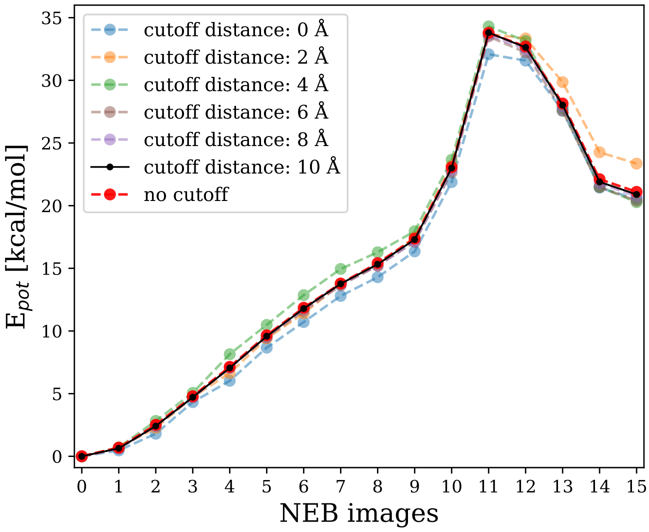

Figure S15: The influence of the cutoff used for the QM–MM electrostatic interactions.

We maintain high accuracy by applying a 10 Å cutoff (black curve) and achieving approximately a 4-fold speedup with respect to those calculations, where the electrostatic interactions between the QM and the MM subsystem were not cut off (red curve). The overall small consequence of fully neglecting the electrostatic interaction with MM atoms (cutoff 0 Å) indicates that the QM region captures the electrostatics of the active site sufficiently well.

6 Benchmark calculations - QM region size

We recomputed the refined NEB path with various QM regions. Water molecules, amino acids or series of amino acids were selected in the QM region is any atoms of these molecules fall within the d distance defined around the reaction center formed by four key atoms from the first reaction step: the two O atoms of Glu305, the O_{wat} atom of the attacking water molecule and P_γ. The cut between the QM and MM regions and the placement of link atoms was made as shown in Figure S1.

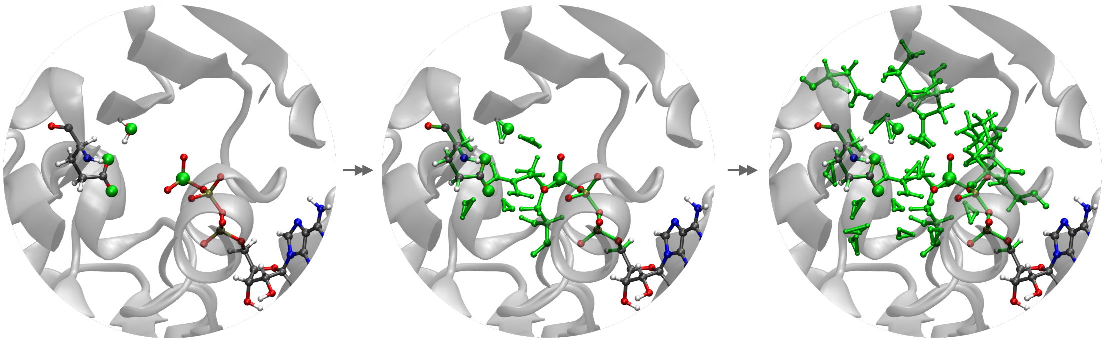

Figure S16: Increasing the QM region around the reaction center defined by the two O atoms of Glu305, O_{wat} and P_γ

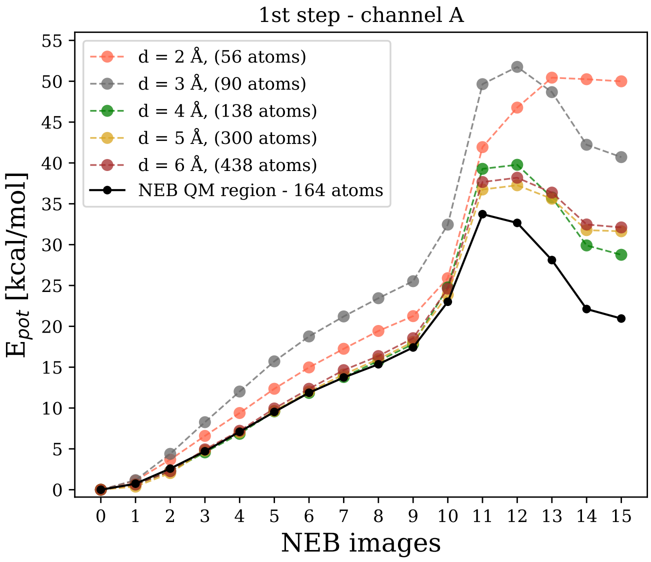

Figure S17: First step: Benchmark calculations for the number of QM atoms used in the QM/MM simulations.

The QM region was systematically increased, including more amino acids and neighboring solvent molecules. All QM regions include the phosphate backbone of the ATP molecule, the nucleophilic water molecule, Glu305, the Mg^{2+} ion, the nucleophilic water and Arg (R359). The smallest QM region (d = 2 Å) does not contain Asn348, without this key residue the minimum corresponding to the intermediate cannot be located. Nevertheless, for cutoff 4 Å(138 atoms) or larger the energy curve is largely conserved, suggesting that the NEB QM region of 164 atoms is a safe choice.

Energy barriers slightly increase for all tested QM regions compared to the QM region used in NEB optimizations. This can be understood by considering that for the test QM regions only single-point energies were computed without re-optimizing the NEB path. The 4.3 kcal/mol difference between the barriers of the converged paths with 138–438 QM atoms and the NEB path with 164 QM atoms can be attributed to the effect of the NEB optimization. The 1000 iterations refer to 1000 steps of the Nudged Elastic Band (NEB) method. During these steps, the reaction path and the images along the path are iteratively refined. The first and last NEB images were frozen during this re-optimization, only focusing on replacing the energy barrier. As shown in Fig. S18 and Fig. S20, this gradual optimization improves the minimum energy path and reduces the energy barrier by approximately 4 kcal/mol, such that the energy gap between the NEB-optimized and recalculated pathways disappears.

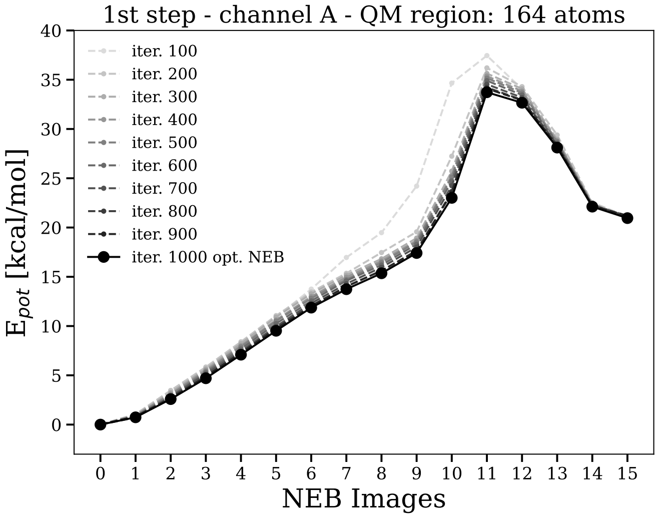

Figure S18: First step: the effect of 1000 NEB iterations on the minimum energy path

Below the same benchmark is shown for the second step: proton transfer from GluE305 to a phosphate oxygen. However, the influence of the QM region on results is less severe than for the first step, and the H^+ transfer process observed in the second step is more local. Therefore, we choose a smaller QM region, consisting of 123 QM atoms.

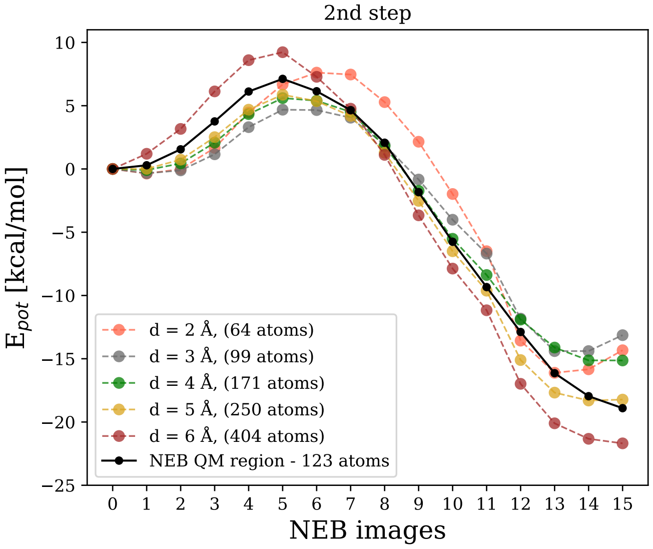

Figure S19: Second step: Benchmark calculations for the number of QM atoms used in the QM/MM simulations.

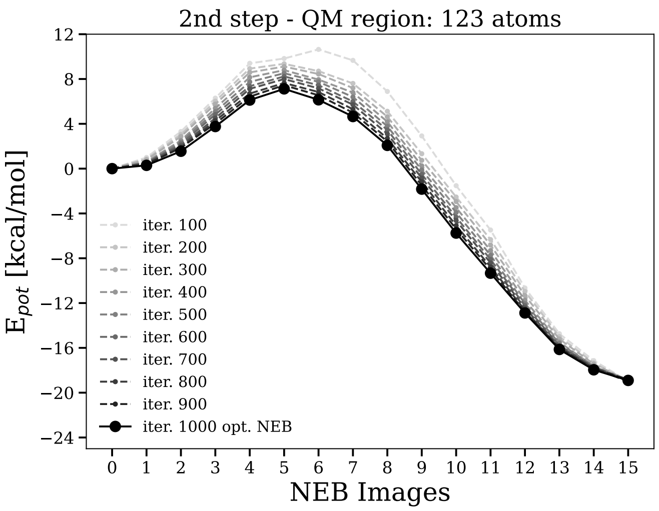

Figure S20: Second step: the effect of 1000 NEB iterations on the minimum energy path.

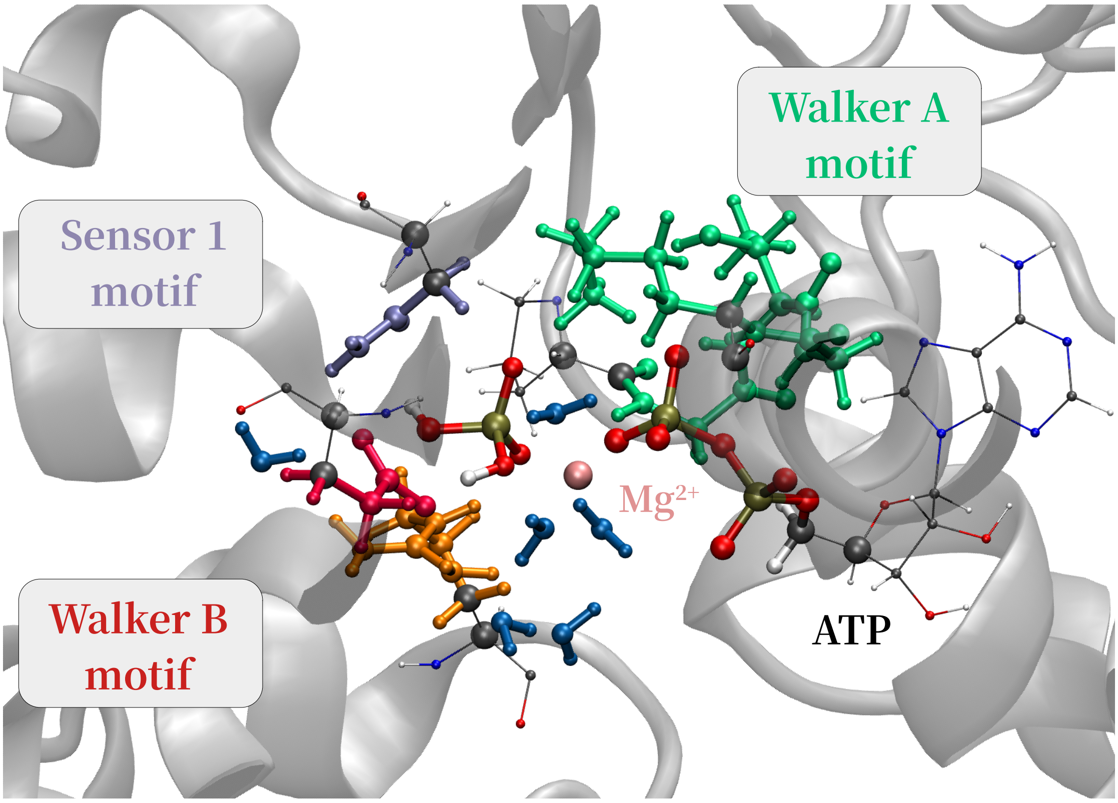

Figure S21: Second step: the QM region consist of 123 atoms - the phosphate backbone of the ATP molecule, the six closest water molecules (blue), Glu305 from the Walker B motif (red), Asn348 from the Sensor 1 motif (purple), Gly248, Thr249, Gly250, and Lys251 from the Walker A motif (green), as well as Arg359 from the adjacent protein subunit (orange). Grey spheres indicate carbon atoms at the QM/MM boundaries, where hydrogen link atoms were introduced; only non-polar C–C bonds were cut to define the QM region.

7 Enhanced sampling using the WTM-eABF method

Reaction free energy profiles were computed from biased QM/MM-MD simulations using the path Well-Tempered Metadynamics extended-system Adaptive Biasing Force (WTM-eABF) algorithm as implemented in the adaptive-sampling package [21]. In the extended-system formulation, the bias forces are not applied directly to the collective variable (CV) but to a fictitious particle. This particle, with a mass of 40 a.u., is coupled to the CV via a harmonic spring, with a thermal width set to 0.01. The CV space is discretized with a bin width of 0.01, and the WTM and ABF forces are accumulated on this grid. The ABF force was scaled up using a linear ramping scheme, where the force applied in each bin was proportional to the number of collected samples with the full force applied only in bins with 200 samples or more. For the metadynamics potential, 4000 K was set as the WTM bias temperature and every 10 fs a new Gaussian hill with a height of 0.1 kJ/mol and with a variance of 0.03 was deposited. The initial height of the Gaussian hills decreases over time due to the well-tempered scaling.

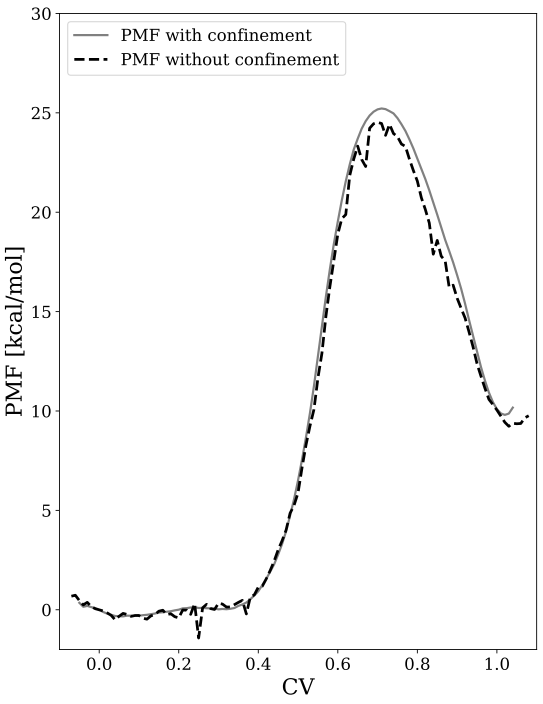

Figure S22: Effect of removing the confinement to the path CV on the PMF profile.

During sampling, harmonic walls with a force constant of 100 kJ/molÅ^2 are applied to confine the CV to the range of interest, thus preventing the system from exploring unwanted configurations. The Multistate Bennett's Acceptance Ratio (MBAR) was used as estimator to compute ensemble averages and PMFs using the unbiased weights of the simulation frames [25, 26]. The MBAR equations are solved self consistently and the

PMF profiles are computed. The PMF can be reconstructed without the bias introduced with the confinement of the path CV, by removing the harmonic confinement potential from the PMF. We apply the geometric path CV definition [18], which requires the selection of an appropriate CV space. While the environment is not confined, the four breaking and forming bond distances (show in Fig. S23) that are included in the CV space of the path CV are forced to stay close to the MEP during the simulation.

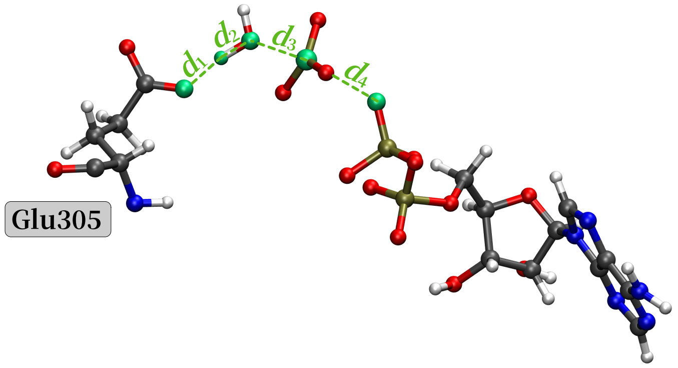

Figure S23: Enhanced sampling of the 1st step in ATP hydrolysis: bond distances employed to build the CV space in the WTM-eABF simulations.

As shown in Fig. S24 and S25, the process starts with the cleavage of the scissile $O_{3B}{-}P_{\gamma}$, followed by the nucleophilic attack of a water molecule, concerted with its deprotonation by Glu. We see that the distance to the path remains relatively constant over the duration of the sampling period, suggesting that the MEB and minimum free energy path (MFEP) are well aligned.

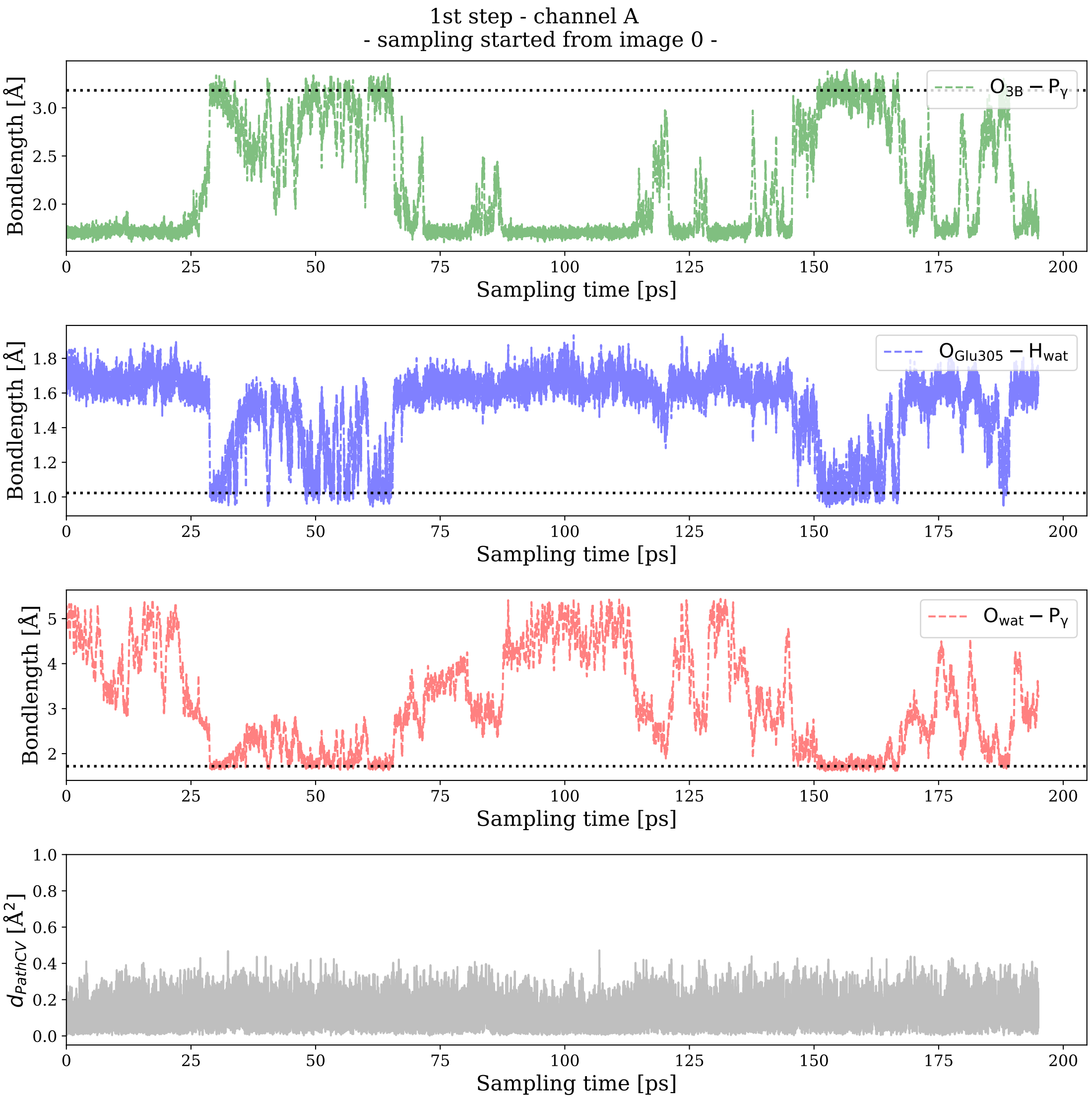

Figure S24: The evolution of key interatomic distances and the distance from the path CV along the sampling time. The WTM-eABF sampling is started from the educt structure (0th NEB image), the dashed lines mark the distances measured in the intermediate structure (15th NEB image), where the H^+ from the water molecule gets transferred to the Glu305, the O_{wat} - P_γ bond is formed and the O_{3B} - P_γ bond is broken.

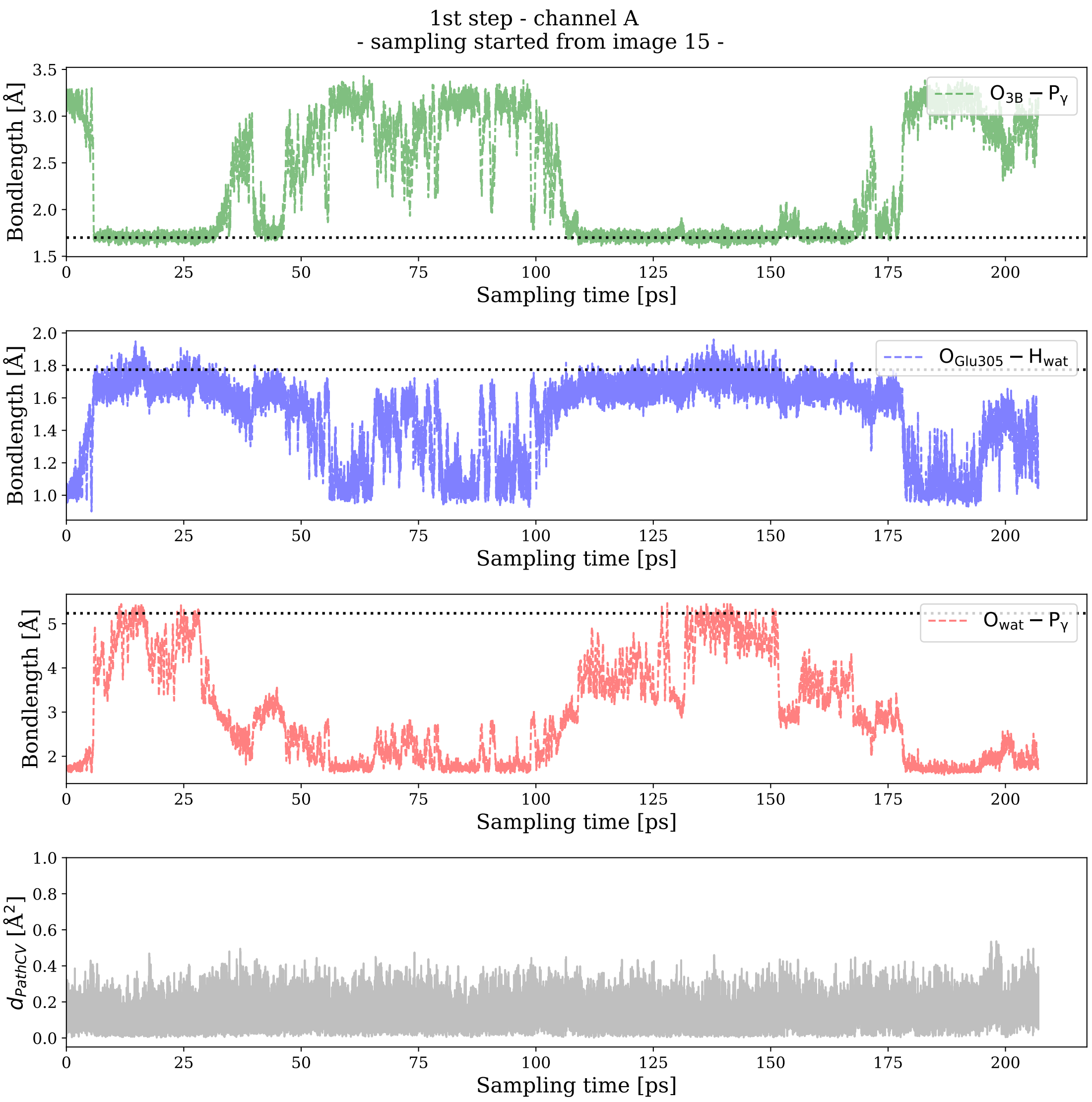

Figure S25: The evolution of key interatomic distances and the distance from the path CV along the sampling time. The WTM-eABF sampling is started from the intermediate structure (15th NEB image), the dashed lines mark the distances measured in the educt structure (0th NEB image), where the O_{3B} - P_γ bond forms again and the water goes away from the terminal phosphate.

8 The educt and the product state - H-bond networks at the binding site

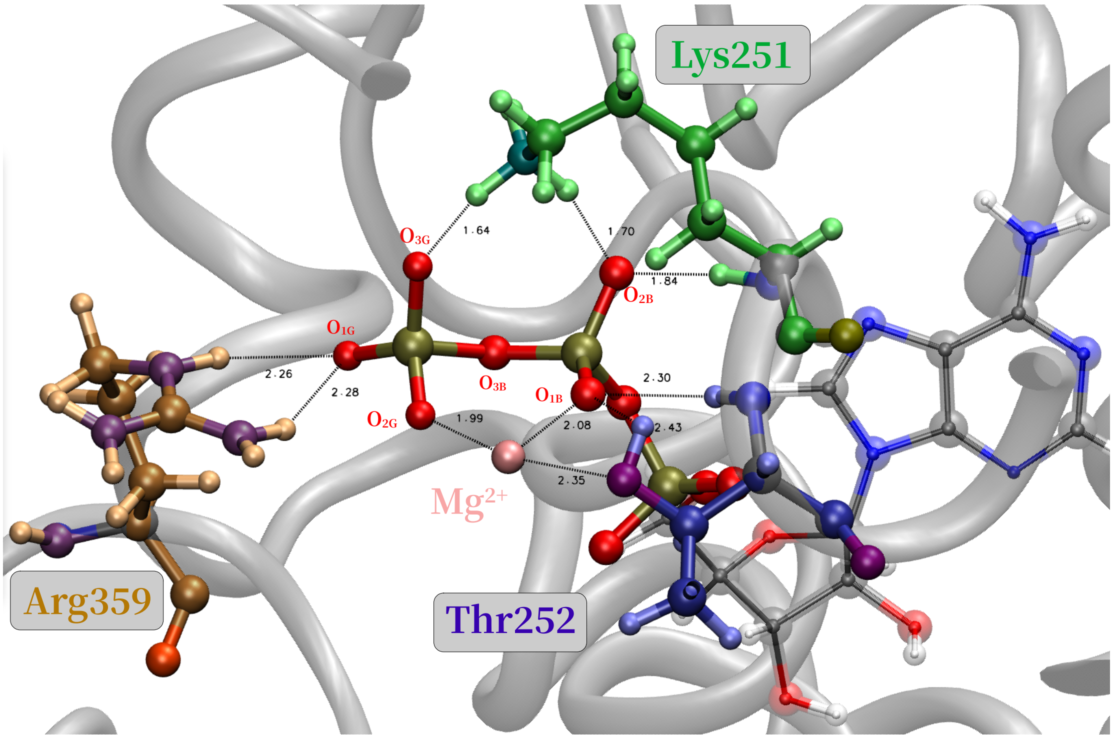

Figure S26: Educt state: H-bond network formed between the substrate, Arg359, Lys251, and Thr252 with the Mg^{2+} coordination sphere.

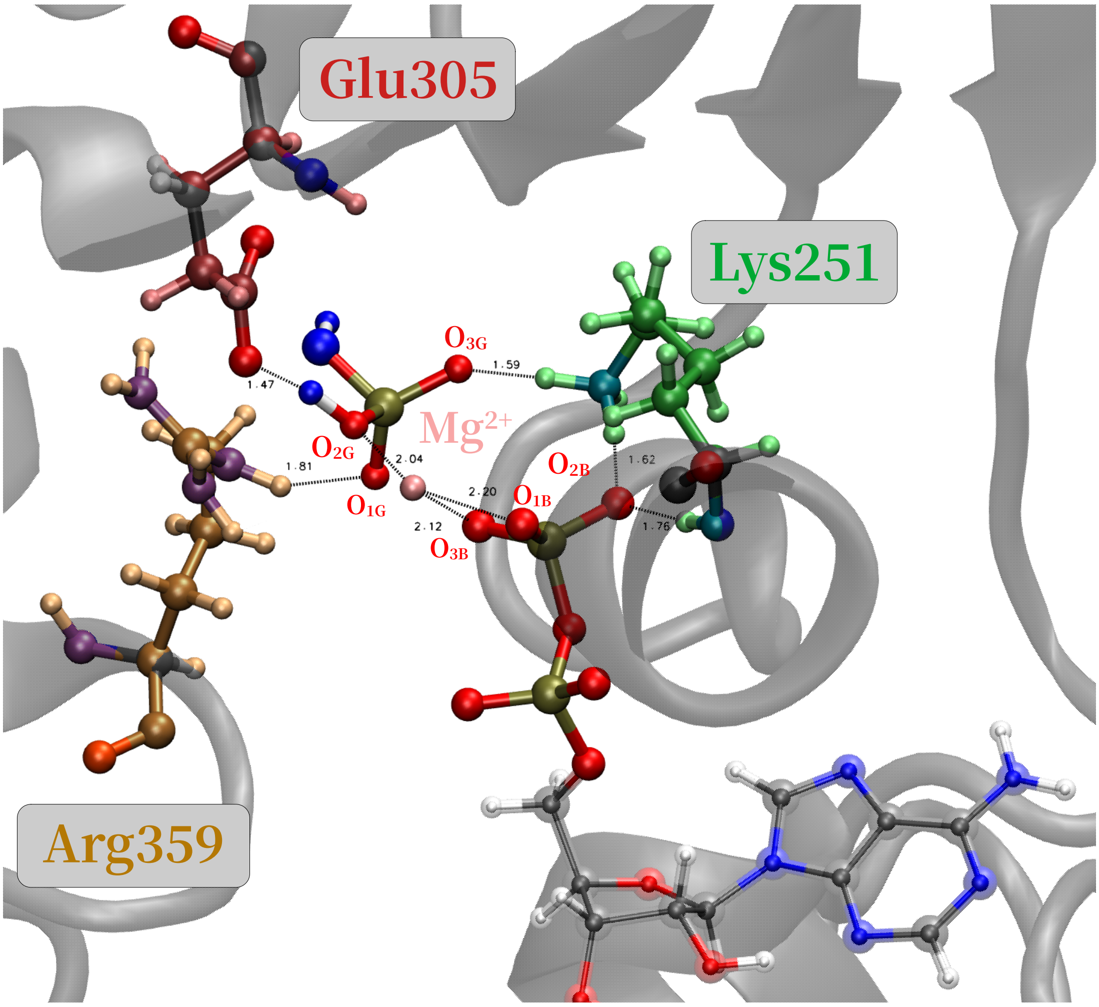

Figure S27: Product state: the Mg^{2+} coordination sphere bridges ADP^{3-} and $H_2PO_4^-$. H-bond network formed between the substrate and key amino acids: Glu305, Arg359, and Lys251.

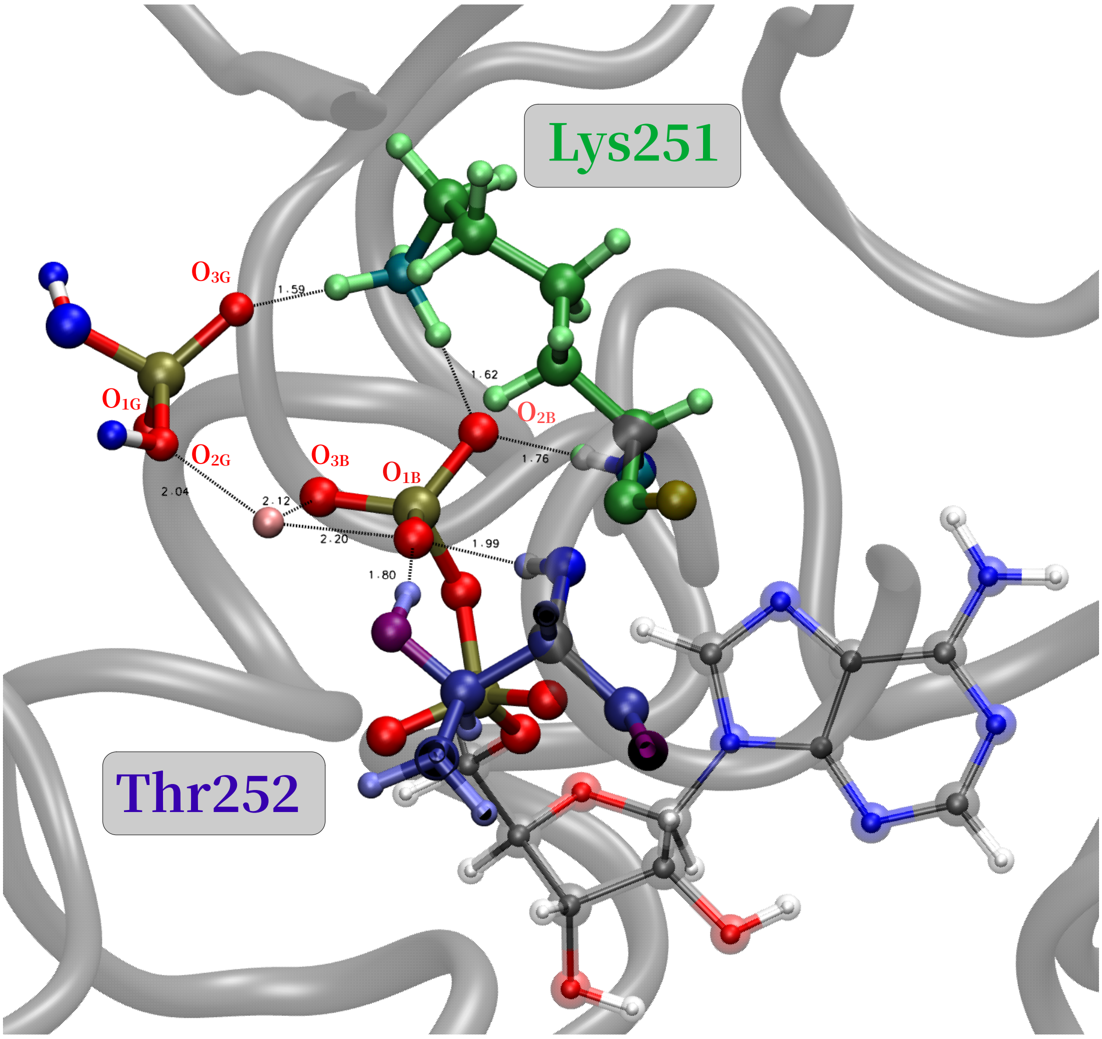

Figure S28: Product state: the Mg^{2+} coordination sphere bridges ADP^{3-} and $H_2PO_4^-$. H-bond network formed between the substrate, Thr252 and Lys251.

In the product state (see Fig. S27), the Mg^{2+} ion is tightly coordinated by oxygen atoms of ADP, P_i (O_{2G}, O_{3B}, O_{1B}) and three water molecules forming an octahedral arrangement.

The NEB pathway that leads to the product state captures two events: the O_{3B} atom enters the Mg^{2+} coordination shell and Thr252 leaves it.

a) b)

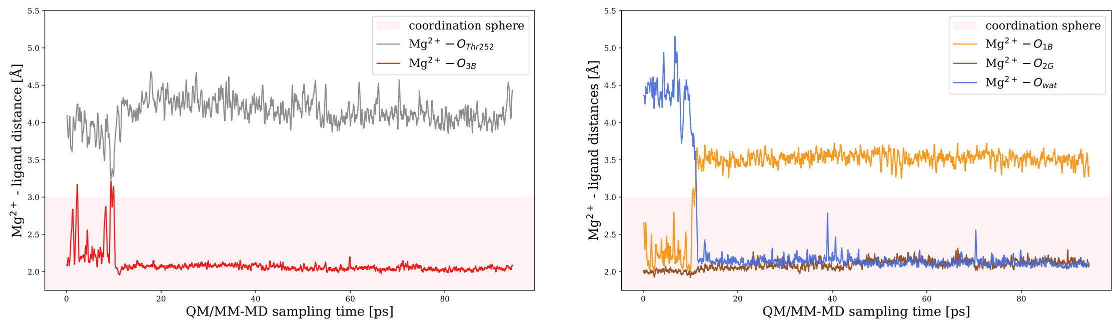

Figure S29: Mg^2+ coordination in the product state over 100 ps.

The 100 ps long unbiased QM/MM-MD simulation of the product state shows that the O_{3B} atom remains strongly coordinated to the Mg^{2+}, while Thr252 stays outside the coordination sphere. The 3-fold Mg^{2+} coordination to the ADP + P_i is stable for 10 ps, after which the O_{1B} atom leaves the coordination shell.

9 DFT NMR calculations

The chemical shift of the P_α atom barely changes as we transition from the educt to the product structure; therefore, this nucleus was used as a reference to convert absolute magnetic shieldings (σ) into chemical shifts (δ). All computed isotropic shieldings along the reaction path were shifted such that the P_α from the educt (0th NEB image of the first step) matches the experimentally measured P_α shift of the ATP at the active site of p97[27].

$$\text{ref} = \sigma_{\text{calc. } (\text{P}_\alpha \text{ in ATP})} + \delta_{\text{exp. } (\text{P}_\alpha \text{ in ATP})}$$

10 Enhanced sampling using the WTM-eABF method - trajectories and histograms for the first reaction step

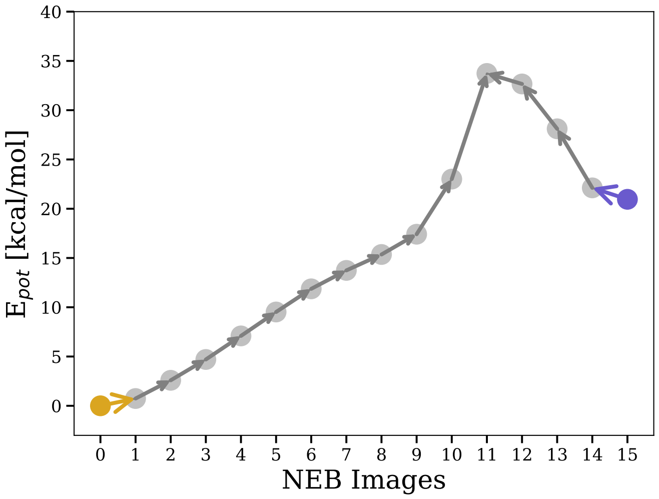

Figure S30: Minimum Energy Pathway from NEB optimizations for channel A. Arrows indicate QM/MM-MD trajectories initiated from NEB images, with bias applied to the path collective variable for sampling. The 0th (gold) and the 15th NEB image (purple) correspond to the educt and the ADP + HPO_4^{2-} intermediate structure.

Sampling data from trajectories started from the 5th and 6th NEB image (Fig. S28 and S29) were excluded from the PMF profile calculation, because these trajectories were trapped in the product state after the first transition.

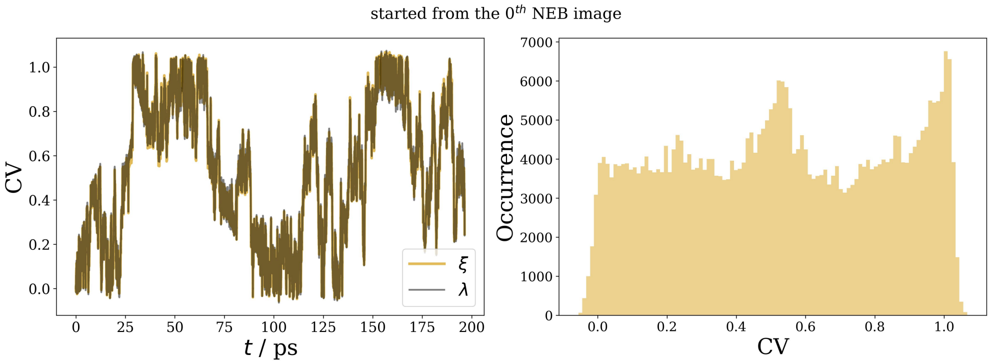

Figure S31: Trajectory and histogram of the path CV for QM/MM-MD sampling started from the NEB image 0.

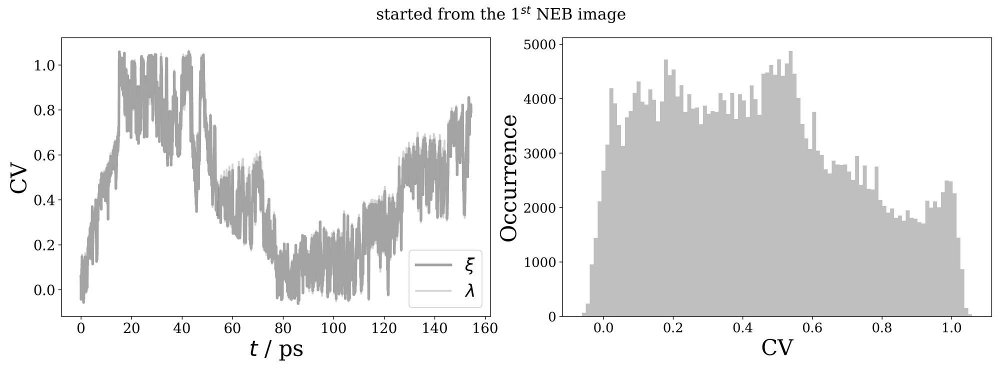

Figure S32: Trajectory and histogram of the path CV for QM/MM-MD sampling started from the NEB image 1.

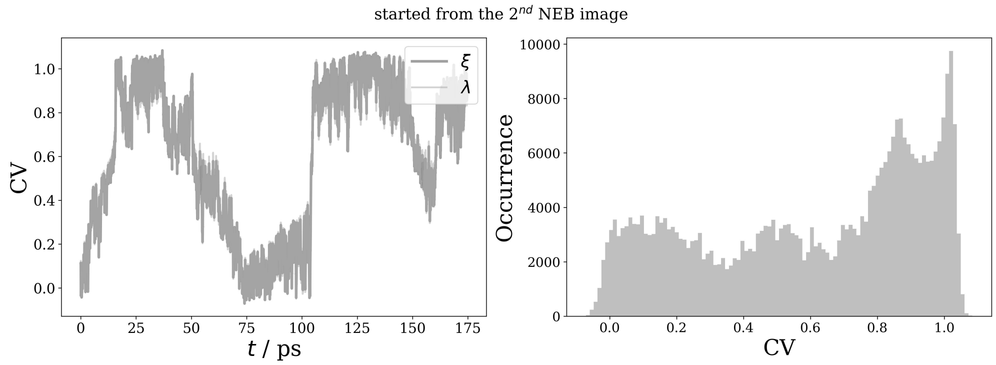

Figure S33: Trajectory and histogram of the path CV for QM/MM-MD sampling started from the NEB image 2.

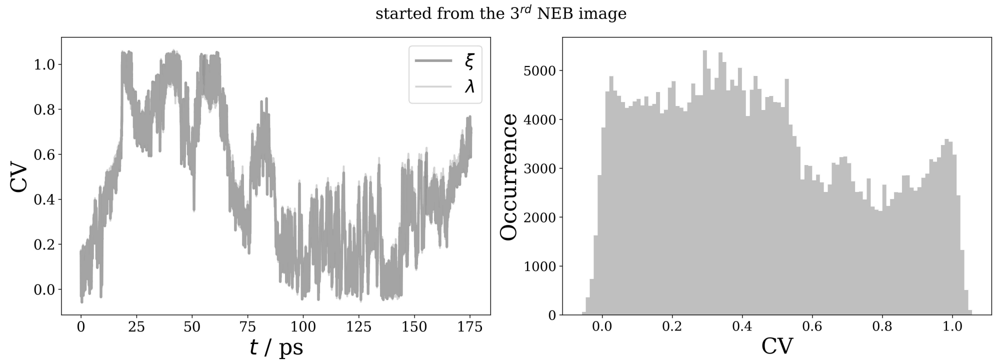

Figure S34: Trajectory and histogram of the path CV for QM/MM-MD sampling started from the NEB image 3.

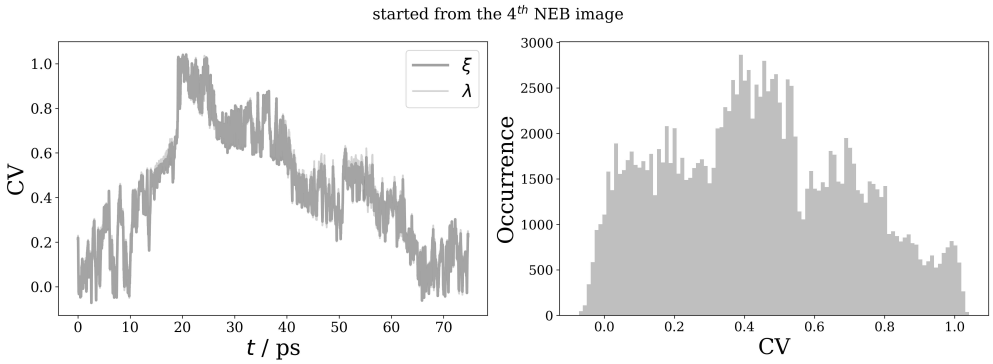

Figure S35: Trajectory and histogram of the path CV for QM/MM-MD sampling started from the NEB image 4.

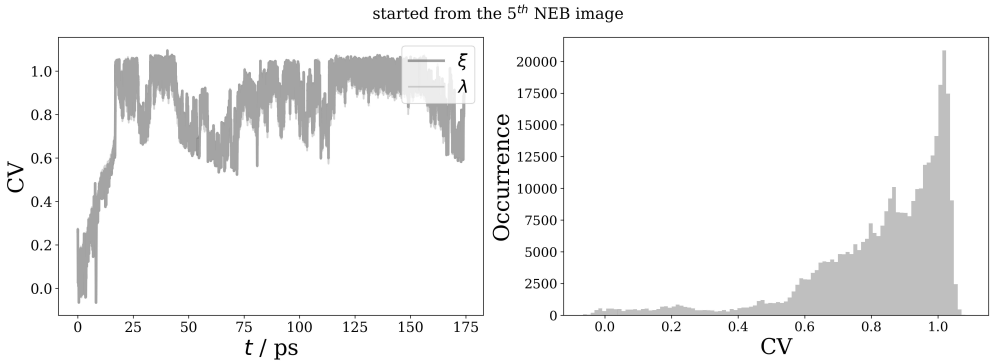

Figure S36: Trajectory and histogram of the path CV for QM/MM-MD sampling started from the NEB image 5.

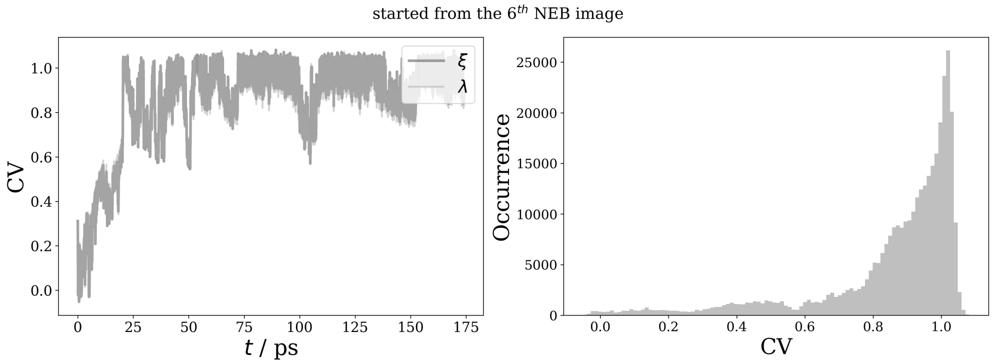

Figure S37: Trajectory and histogram of the path CV for QM/MM-MD sampling started from the NEB image 6.

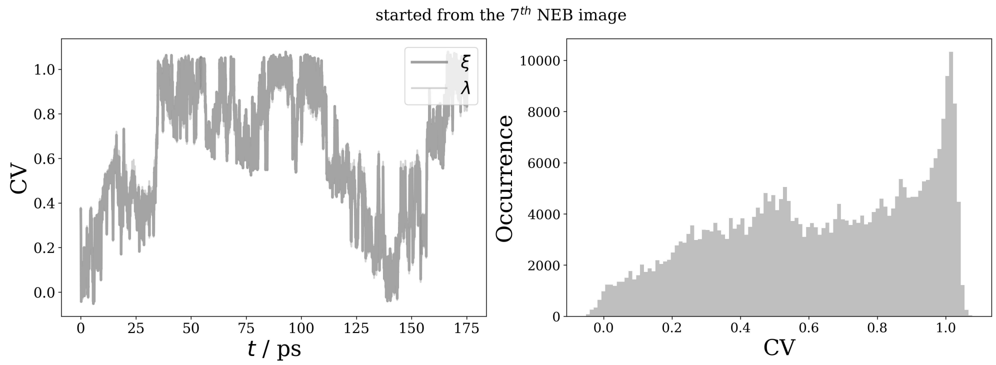

Figure S38: Trajectory and histogram of the path CV for QM/MM-MD sampling started from the NEB image 7.

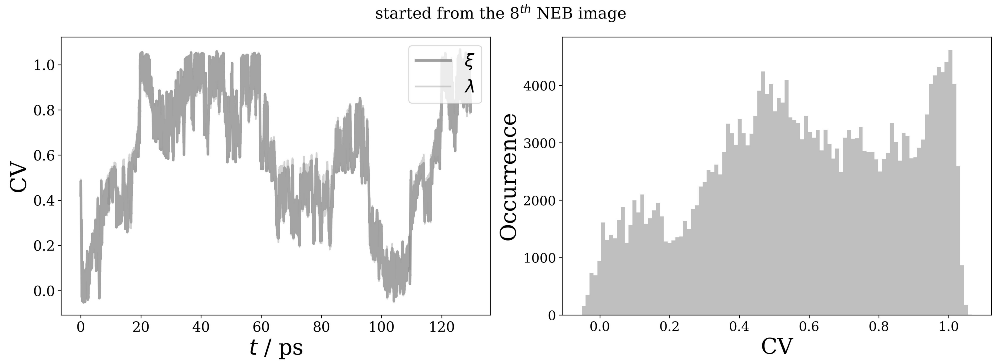

Figure S39: Trajectory and histogram of the path CV for QM/MM-MD sampling started from the NEB image 8.

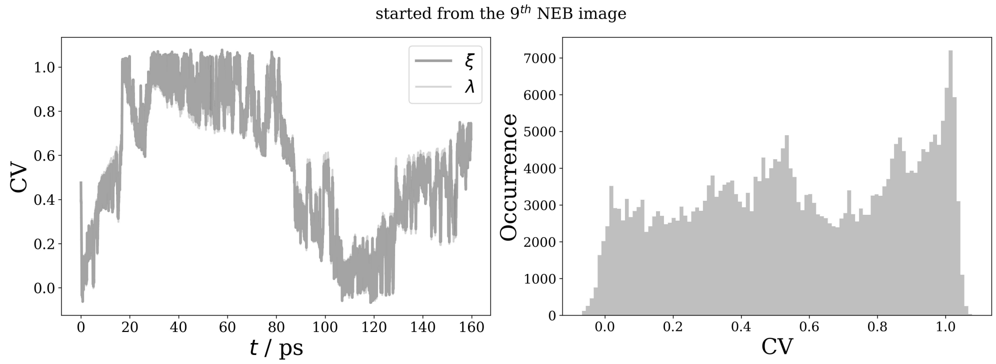

Figure S40: Trajectory and histogram of the path CV for QM/MM-MD sampling started from the NEB image 9.

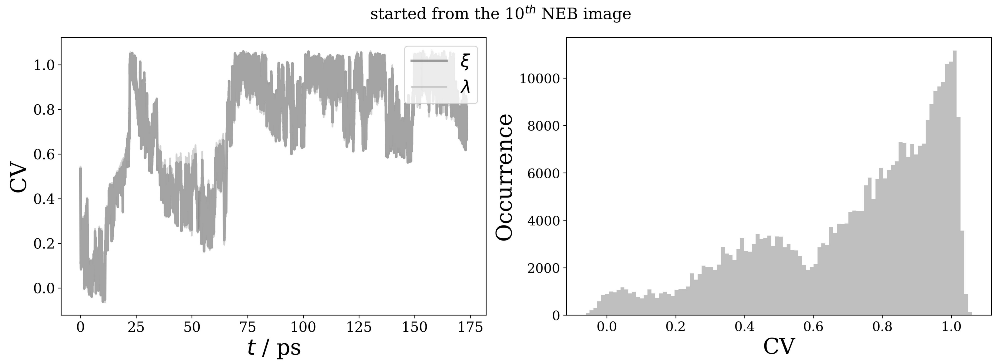

Figure S41: Trajectory and histogram of the path CV for QM/MM-MD sampling started from the NEB image 10.

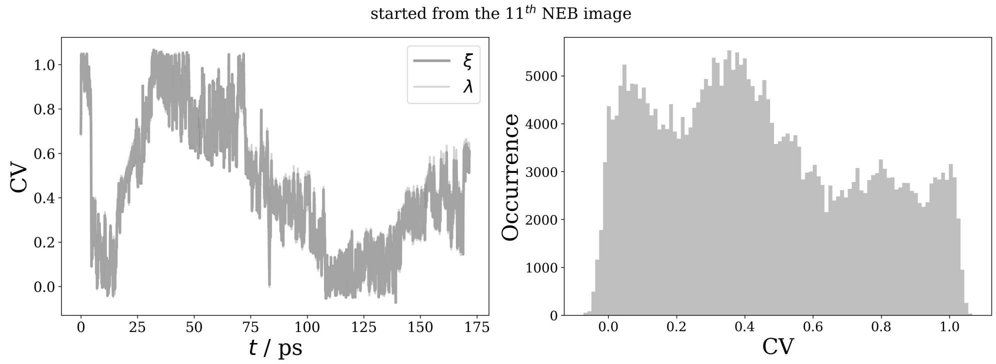

Figure S42: Trajectory and histogram of the path CV for QM/MM-MD sampling started from the NEB image 11.

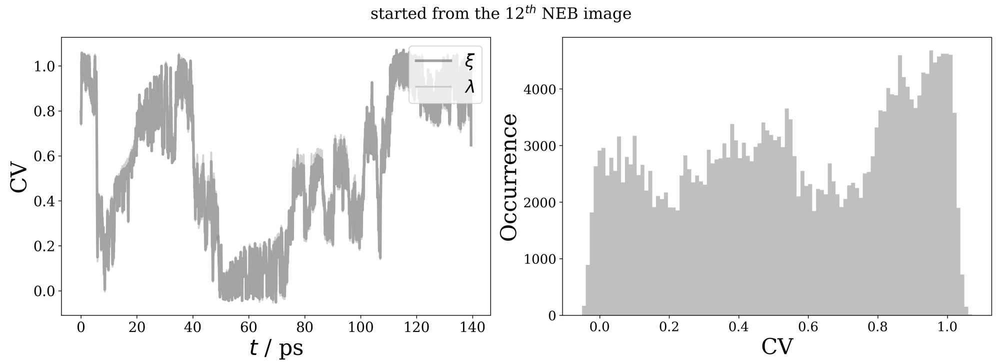

Figure S43: Trajectory and histogram of the path CV for QM/MM-MD sampling started from the NEB image 12.

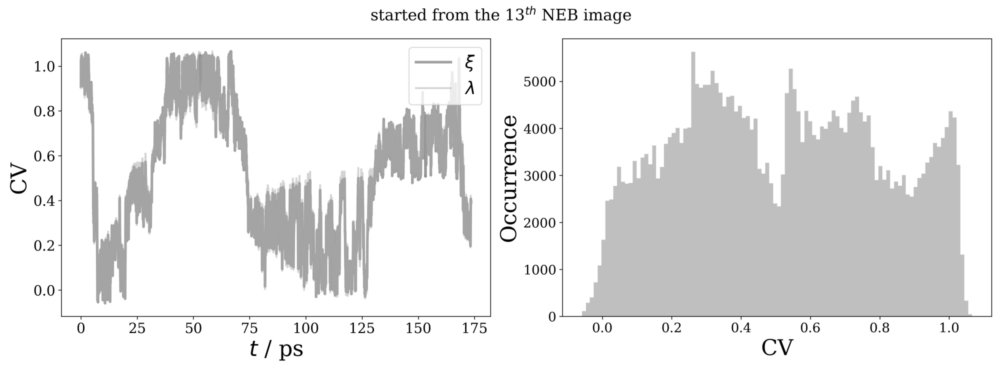

Figure S44: Trajectory and histogram of the path CV for QM/MM-MD sampling started from the NEB image 13.

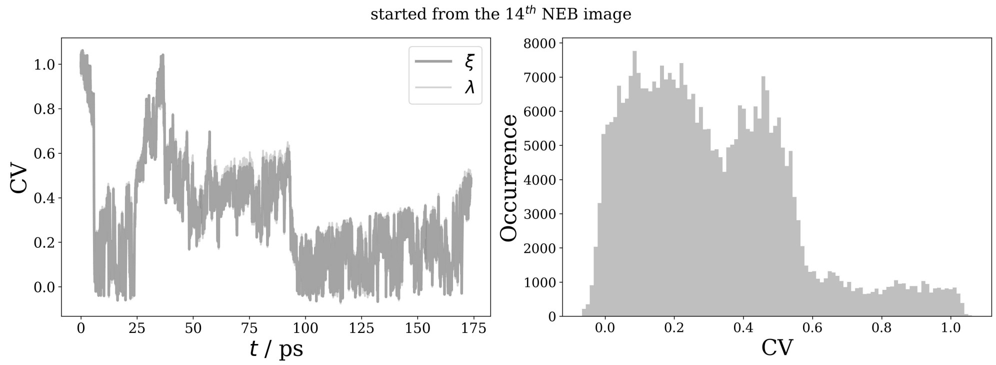

Figure S45: Trajectory and histogram of the path CV for QM/MM-MD sampling started from the NEB image 14.

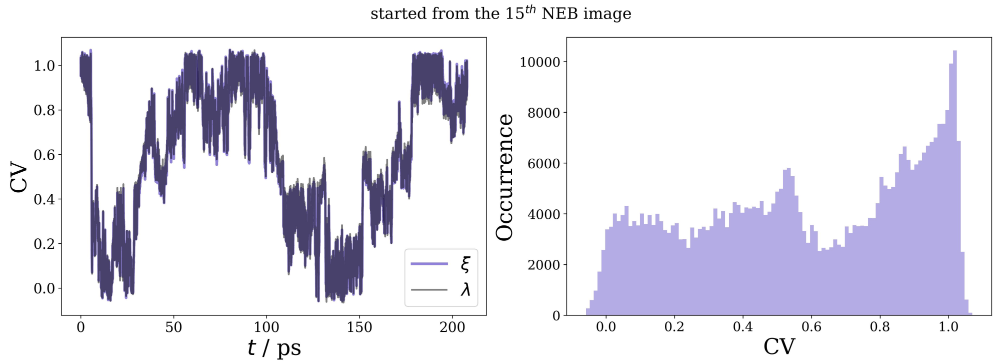

Figure S46: Trajectory and histogram of the path CV for QM/MM-MD sampling started from the NEB image 15.

11 Evaluation of the PMF profile uncertainties

Uncertainties associated with the computed free energy profiles are analyzed in the following ways. Firstly, the variation of the PMF profile as obtained from individual trajectories is computed (shaded region in Figure S47).

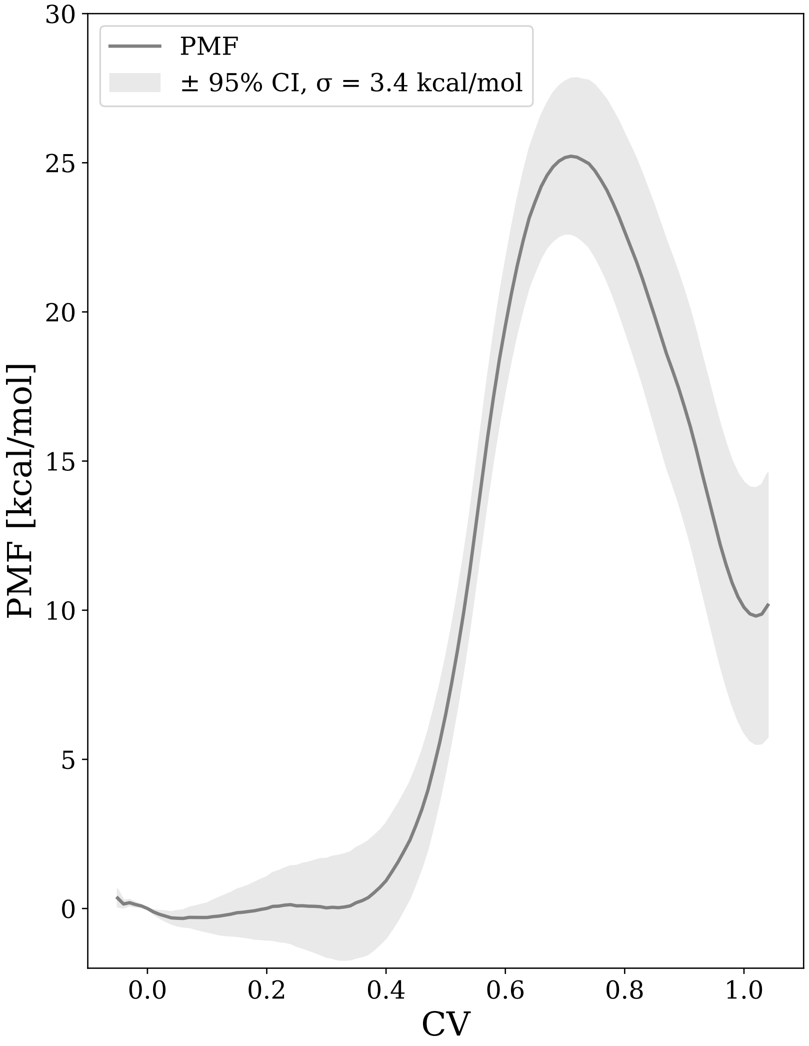

Figure S47: PMF profile uncertainty predicted from individual trajectories. The shaded region illustrates the 95% confidence interval.

Secondly, we use a subsample bootstrap approach to create $N_{sample} = 100$ datasets, each containing as many data points as a single trajectory (200 ps), and recalculate the PMF using the same MBAR protocol.

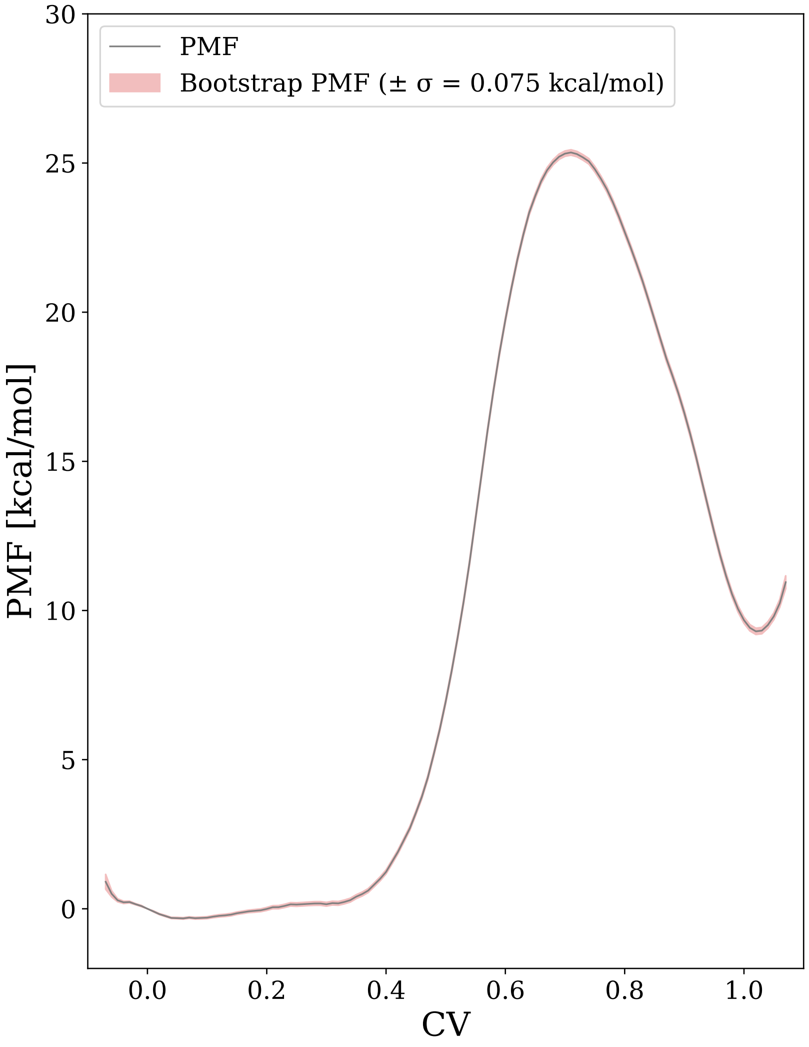

Figure S48: PMF computed from 100 bootstrap subsamples.

In this second approach, the uncertainty can be estimated as the standard deviation across the resulting ensemble of PMFs (Figure S48). The obtained error bars are vanishingly small, reflecting the statistical robustness of the PMF estimation from the global data.

References

(1) Kussmann, J.; Ochsenfeld, C. Pre-selective screening for matrix elements in linear-scaling exact exchange calculations. *J. Chem. Phys.* **2013**, *138*, 134114.

(2) Kussmann, J.; Ochsenfeld, C. Preselective screening for linear-scaling exact exchange-gradient calculations for graphics processing units and general strong-scaling massively parallel calculations. *J. Chem. Theory Comput.* **2015**, *11*, 918–922.

(3) Kussmann, J.; Ochsenfeld, C. Hybrid CPU/GPU integral engine for strong-scaling ab initio methods. *J. Chem. Theory Comput.* **2017**, *13*, 3153–3159.

(4) Grimme, S.; Brandenburg, J. G.; Bannwarth, C.; Hansen, A. Consistent structures and interactions by density functional theory with small atomic orbital basis sets. *J. Chem. Phys.* **2015**, *143*.

(5) Laqua, H.; Thompson, T. H.; Kussmann, J.; Ochsenfeld, C. Highly efficient, linear-scaling seminumerical exact-exchange method for graphic processing units. *J. Chem. Theory Comput.* **2020**, *16*, 1456–1468.

(6) Laqua, H.; Kussmann, J.; Ochsenfeld, C. Accelerating semi-numerical Fock-exchange calculations using mixed single-and double-precision arithmetic. *J. Chem. Phys.* **2021**, *154*, 214116.

(7) Laqua, H.; Dietschreit, J. C.; Kussmann, J.; Ochsenfeld, C. Accelerating Hybrid Density Functional Theory Molecular Dynamics Simulations by Seminumerical Integration, Resolution-of-the-Identity Approximation, and Graphics Processing Units. *J. Chem. Theory Comput.* **2022**, *18*, 6010–6020.

(8) Kussmann, J.; Laqua, H.; Ochsenfeld, C. Highly efficient resolution-of-identity density functional theory calculations on central and graphics processing units. *J. Chem. Theory Comput.* **2021**, *17*, 1512–1521.

(9) Shein, M.; Hitzenberger, M.; Cheng, T. C.; Rout, S. R.; Leitl, K. D.; Sato, Y.; Zacharias, M.; Sakata, E.; Schütz, A. K. Characterizing ATP processing by the AAA+ protein p97 at the atomic level. *Nat. Chem.* **2024**, *16*, 363–372.

(10) Szántó, J. K.; Dietschreit, J. C.; Shein, M.; Schütz, A. K.; Ochsenfeld, C. Systematic QM/MM Study for Predicting 31P NMR Chemical Shifts of Adenosine Nucleotides in Solution and Stages of ATP Hydrolysis in a Protein Environment. *J. Chem. Theory Comput.* **2024**, *20*, 2433–2444.

(11) Kästner, J.; Carr, J. M.; Keal, T. W.; Thiel, W.; Wander, A.; Sherwood, P. DL-FIND: An open-source geometry optimizer for atomistic simulations. *J. Phys. Chem. A* **2009**, *113*, 11856–11865.

(12) Lu, Y.; Farrow, M. R.; Fayon, P.; Logsdail, A. J.; Sokol, A. A.; Catlow, C. R. A.; Sherwood, P.; Keal, T. W. Open-Source, python-based redevelopment of the ChemShell multiscale QM/MM environment. *J. Chem. Theory Comput.* **2018**, *15*, 1317–1328.

(13) Hulm, A.; Lemke, Y.; Johannes, D.; Glinkina, L.; Stan-Bernhardt, A. adaptive_sampling, https://github.com/ochsenfeld-lab/adaptive_sampling.

(14) Tang, W. K.; Xia, D. Altered intersubunit communication is the molecular basis for functional defects of pathogenic p97 mutants. *J. Biol. Chem.* **2013**, *288*, 36624–36635.

(15) Ranaghan, K. E.; Mulholland, A. J. Investigations of enzyme-catalysed reactions with combined quantum mechanics/molecular mechanics (QM/MM) methods. *Int. Rev. Phys. Chem.* **2010**, *29*, 65–133.

(16) Lonsdale, R.; Harvey, J. N.; Mulholland, A. J. A practical guide to modelling enzyme-catalysed reactions. *Chem. Soc. Rev.* **2012**, *41*, 3025–3038.

(17) Henkelman, G.; Jónsson, H. Improved tangent estimate in the nudged elastic band method for finding minimum energy paths and saddle points. *J. Chem. Phys.* **2000**, *113*, 9978–9985.

(18) Díaz Leines, G.; Ensing, B. Path finding on high-dimensional free energy landscapes. *Phys. Rev. Lett.* **2012**, *109*, 020601.

(19) Fu, H.; Zhang, H.; Chen, H.; Shao, X.; Chipot, C.; Cai, W. Zooming across the free-energy landscape: shaving barriers, and flooding valleys. *J. Phys. Chem. Lett.* **2018**, *9*, 4738–4745.

(20) Fu, H.; Shao, X.; Cai, W.; Chipot, C. Taming rugged free energy landscapes using an average force. *Acc. Chem. Res.* **2019**, *52*, 3254–3264.

(21) Hulm, A.; Ochsenfeld, C. Improved Sampling of Adaptive Path Collective Variables by Stabilized Extended-System Dynamics. *J. Chem. Theory Comput.* **2023**, *19*, 9202–9210.

(22) Neese, F. Software update: The ORCA program system—Version 5.0. *Wiley Interdiscip. Rev. Comput. Mol. Sci.* **2022**, *12*, e1606.

(23) Müller, M.; Hansen, A.; Grimme, S. ωB97X-3c: A composite range-separated hybrid DFT method with a molecule-optimized polarized valence double-ζ basis set. *J. Chem. Phys.* **2023**, *158*.

(24) Riplinger, C.; Sandhoefer, B.; Hansen, A.; Neese, F. Natural triple excitations in local coupled cluster calculations with pair natural orbitals. *J. Chem. Phys.* **2013**, *139*.

(25) Shirts, M. R.; Chodera, J. D. Statistically optimal analysis of samples from multiple equilibrium states. *J. Chem. Phys.* **2008**, *129*, 124105.

(26) Hulm, A.; Dietschreit, J. C.; Ochsenfeld, C. Statistically optimal analysis of the extended-system adaptive biasing force (eABF) method. *J. Chem. Phys.* **2022**, *157*.

(27) Rydzek, S.; Shein, M.; Bielytskyi, P.; Schütz, A. K. Observation of a transient reaction intermediate illuminates the mechanochemical cycle of the AAA-ATPase p97. *J. Am. Chem. Soc.* **2020**, *142*, 14472–14480.
